# Galectin-1 dimers can scaffold Raf-effectors to increase H-ras nanoclustering

**DOI:** 10.1038/srep24165

**Published:** 2016-04-18

**Authors:** Olga Blaževitš, Yonatan G. Mideksa, Maja Šolman, Alessio Ligabue, Nicholas Ariotti, Hossein Nakhaeizadeh, Eyad K. Fansa, Anastassios C. Papageorgiou, Alfred Wittinghofer, Mohammad R. Ahmadian, Daniel Abankwa

**Affiliations:** 1 Turku Centre for Biotechnology, Åbo Akademi University, Tykistökatu 6B, 20520 Turku, Finland; 2 Institute for Molecular Bioscience, The University of Queensland, St. Lucia, QLD 4072, Australia; 3 Institute of Biochemistry and Molecular Biology II, Medical Faculty, Heinrich-Heine-University, Düsseldorf, Germany; 4 Max Planck Institute for Molecular Physiology, 44227 Dortmund, Germany

## Abstract

Galectin-1 (Gal-1) dimers crosslink carbohydrates on cell surface receptors. Carbohydrate-derived inhibitors have been developed for cancer treatment. Intracellularly, Gal-1 was suggested to interact with the farnesylated C-terminus of Ras thus specifically stabilizing GTP-H-ras nanoscale signalling hubs in the membrane, termed nanoclusters. The latter activity may present an alternative mechanism for how overexpressed Gal-1 stimulates tumourigenesis. Here we revise the current model for the interaction of Gal-1 with H-ras. We show that it indirectly forms a complex with GTP-H-ras via a high-affinity interaction with the Ras binding domain (RBD) of Ras effectors. A computationally generated model of the Gal-1/C-Raf-RBD complex is validated by mutational analysis. Both cellular FRET as well as proximity ligation assay experiments confirm interaction of Gal-1 with Raf proteins in mammalian cells. Consistently, interference with H-rasG12V-effector interactions basically abolishes H-ras nanoclustering. In addition, an intact dimer interface of Gal-1 is required for it to positively regulate H-rasG12V nanoclustering, but negatively K-rasG12V nanoclustering. Our findings suggest stacked dimers of H-ras, Raf and Gal-1 as building blocks of GTP-H-ras-nanocluster at high Gal-1 levels. Based on our results the Gal-1/effector interface represents a potential drug target site in diseases with aberrant Ras signalling.

The small GTPase Ras is a major signal transducer, which relays mitogenic signals across the membrane into the cell. Its central role during cell proliferation and differentiation is underscored by the high frequency of Ras mutations in cancer^1^. GTP-loaded Ras adopts different conformations, enabling it to interact with downstream effector proteins, such as the Raf kinases. The three Ras isoforms, H-, N- and K-ras, are frequently mutated on codons 12, 13 and 61 in cancer^1^. These mutations render Ras insensitive to GTPase activating protein (GAP) mediated GTP hydrolysis. As a result, Ras is left constitutively GTP-bound and therefore active, as Ras itself is a poor GTPase^2^. Two splice isoforms of K-ras4A and K-ras4B, are both expressed in cancer^3^. However, historically, K-ras4B (hereafter K-ras) has received most attention. While K-ras is considered the most significant Ras isoform, due to its association with many aggressive cancers, recent insight into the origin of cancer cells warrants further investigation of the specific functions of all three Ras isoforms^4^.

Ras proteins are highly similar in sequence and vary mostly in their C-terminal hypervariable region (hvr). This part undergoes post-translational farnesylation and palmitoylation (the latter for H- and N-ras) allowing Ras to dynamically insert into cellular membranes^5^. Ras is actively transported to the plasma membrane, where it is further organised into nanoscale signalling hubs, called nanoclusters. A Ras nanocluster comprises 6–8 Ras proteins, which in the case of the active Ras becomes transiently immobilized^6^,^7^,^8^. Nanoclusters are the exclusive sites of effector recruitment thus constituting highly dynamic epicentres of the Ras signalling cascade^9^,^10^. Nanoclustering is driven by the C-terminal membrane anchor of Ras, which also largely dictates their lateral segregation into isoform specific nanoclusters^11^,^12^. Importantly, these features are shared with Ras dimers, which appear to constitute the smallest ‘nanocluster’^13^. Thus laterally segregated, Ras isoform specific nanoscale oligomeric clusters constitute an important experimental observable that correlates with the structural and functional divergence of the different Ras proteins and the emergence of Ras signalling complexes.

Only very few endogenous regulators of Ras nanoclustering, so called nanocluster scaffolds, are known. These include galectin-3^14^, nucleophosmin^15^, caveolae^16^ and GTP-H-ras^17^ for K-ras, and galectin-1 (Gal-1)^9^,^10^,^18^ for GTP-H-ras. Amongst these, Gal-1 is the best-characterised nanocluster scaffold. Gal-1 is a prototypic member of the family of β-galactoside binding lectins (galectins), of which there are 15 in humans^19^. Galectins are small (*ca.* 15 kDa for a prototypical single carbohydrate binding monomer) proteins formed by two antiparallel β-sheets. The carbohydrate is coordinated by the highly conserved carbohydrate binding site^20^. Gal-1 localizes to the cytoplasm and nucleus and is also secreted by an unknown pathway^21^. Due to oxidation of free cysteines in the protein, secreted Gal-1 loses lectin-binding activity, unless it binds as a dimer to glycoconjugates on the outside of the cell^22^,^23^.

Gal-1 is upregulated in many tumours and associated with more progressive and invasive cancer stages^24^,^25^, as well as radio-^26^ and drug-resistance^27^,^28^. A number of inhibitors against galectins are under development, which are typically competitors of the natural carbohydrate ligands^29^,^30^,^31^,^32^.

The current model for the nanocluster scaffolding activity of Gal-1 suggests that it directly binds to the C-terminal farnesyl of active H-ras to modulate its intracellular membrane organisation^33^,^34^. Augmented nanoclustering then increases effector recruitment thus potentiating MAPK signalling output^9^,^35^,^36^. Importantly, the Gal-1/GTP-H-ras interaction is suggested to be the target of the anti-Ras drug Salirasib (developed as farnesylthiosalicylic acid, FTS), which is currently assessed preclinically and in clinical trials for the treatment of cancer^37^.

Förster Resonance Energy Transfer (FRET) was amongst the first methods to be used to study the nanoscale membrane organisation of proteins in the intact cell^13^. In FRET a donor fluorophore transfers part of its energy to an acceptor fluorophore, when they are as close as <10 nm, such as in nanoclusters. In addition, FRET is frequently used to measure protein-protein interactions and conformational changes^38^. In particular fluorescence lifetime imaging microscopy (FLIM) allows the fast and precise quantitation of FRET^39^. In FLIM-FRET, FRET is observed by the decrease of the donor fluorescence lifetime.

Using a wide spectrum of quantitative *in vitro* and cellular assays as well as computational modelling we show here that Gal-1 does not directly bind to H-ras, but instead to the Ras binding domain (RBD) of Ras effectors, such as Raf. This explains how Gal-1 specifically recognizes active (GTP-)Ras. We furthermore show that while Gal-1 positively regulates GTP-H-ras nanoclustering, it has the opposite effect on GTP-K-ras and both of these activities depend on its intact dimer interface. We present an entirely revised model of the mechanism of action of Gal-1 as a nanocluster scaffold and briefly discuss implications for Gal-1 and Ras drug development.

## Results

### Galectin-1 does not directly bind to H-ras

Galectin-1 (Gal-1) specifically stabilizes nanoclusters of active (GTP-)H-ras, thus augmenting H-ras signalling output^10^,^36^. According to the current model, Gal-1 interacts directly with the farnesyl-moiety of Ras in a way analogous to the binding of RhoGDI to the geranylgeranyl-moiety of Rho proteins^33^. Gal-1 should therefore effectively function as a chaperone that solubilizes farnesylated proteins and by a structurally unknown mechanism also as a nanocluster scaffold. The stage II (ClinicalTrials.gov identifier NCT00531401) drug farnesylthiosalicylic acid (Salirasib) is a farnesyl-derivative, which was undergoing clinical trials for lung cancer treatment^40^. It was initially found to disrupt the H-ras/Gal-1-interaction in biochemical experiments^35^. It is therefore of high translational relevance to understand this interaction in molecular-mechanistic detail.

In cellular FRET experiments we observed that H-ras and Gal-1 are in a complex in the cytoplasm, even after farnesylation of Ras is blocked by statin treatment or mutation of the cysteine in the C-terminal CAAX-box of Ras that normally undergoes farnesylation (Supplementary Fig. 1A,B). Likewise, the recently described mutation K28T (K29T according to our numbering) on Gal-1, which was shown to block farnesyl-recognition^41^, did not abolish FRET between H-rasG12V and Gal-1 (Supplementary Fig. 1B). These observations were at variance with the previously proposed farnesyl-dependent interaction model by Rotblat *et al.*^33^.

**Figure 1:**
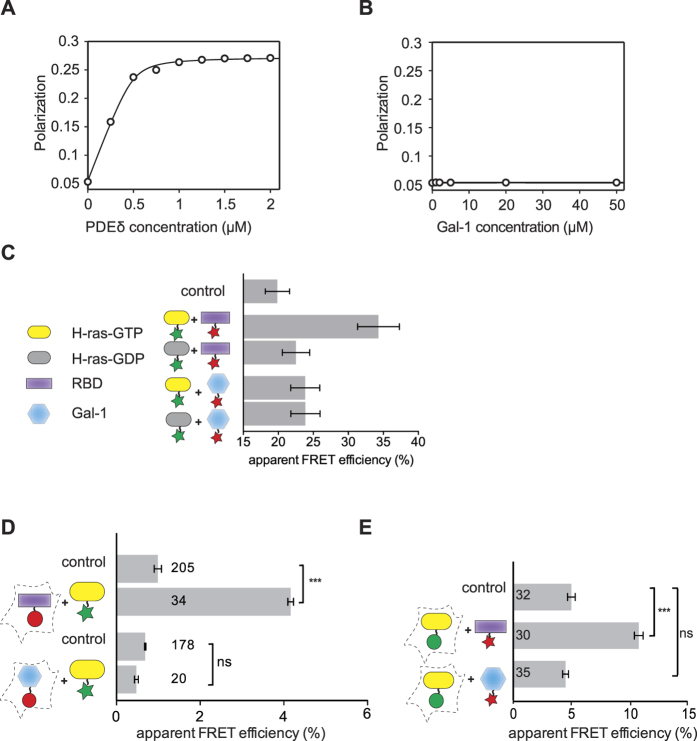
Galectin-1 neither binds to a farnesylated Ras-peptide nor to H-ras in solution experiments. (**A,B**) Fluorescence polarization binding assay of 0.25 μM fluorescein labelled and farnesylated Rheb peptide titrated with increasing concentrations of (**A**) the farnesyl-binding protein PDEδ or (**B**) purified Gal-1. (**C**) Sensitized acceptor FRET binding experiment of 250 nM H-ras and 250 nM Gal-1 or 250 nM C-Raf–RBD (RBD) fluorescently labelled using the ACP-tag technology. The legend to the left shows interaction partners schematically. H-ras was either GTPγS (GTP) or GDP loaded, as indicated. Fluorescent labelling substrates, coenzyme A (CoA)-linked ATTO-488 as a FRET-donor and DY-547 as FRET-acceptor, are represented by green and red stars, respectively. Control sample was a 1:1 mix of fluorescent labelling substrates each at 100 nM. Error bars indicate measurement error. (**D,E**) Interaction between H-ras and Gal-1 or the C-Raf-RBD (RBD) as indicated by the legend in (**C**) was determined by FLIM-FRET. Purified proteins as in (**C**) were incubated with fluorescent protein tagged proteins derived from BHK21 cell lysates (indicated with dotted cell outline). (**D**) Proteins from cell lysates were mRFP-tagged (red circle). (**E**) H-rasG12V labelled with mGFP (green circle) from lysates was used. Control is either mRFP-tagged C-Raf-RBD (upper column) or Gal-1 (lower column) incubated with 1 μM of CoA-488 in (**D**) and mGFP-H-rasG12V incubated with 1 μM of CoA-547 label in (**E**). (**C–E**) Binding of GTP-H-ras and the C-Raf-RBD served as a positive control. (**D,E**) Plotted values correspond to the mean ± SEM from three independent biological repeats. Numbers inside or above the bars indicate total number of imaged regions. The Methods section describes indicated statistical comparisons (ns, non-significant; ***p < 0.001).

In an effort to explain this contradictory observation, we scrutinized the structural basis of the specific Gal-1/H-ras interaction. We first studied their complex formation in solution outside of the membrane. In order to test whether Gal-1 could indeed bind farnesylated Ras-proteins, we employed a fluorescence polarization binding assay that was recently used to demonstrate the ability of the Ras trafficking chaperone PDEδ to solubilize farnesylated proteins^42^. Incubation of a fluorescently labelled peptide derived from the Ras-family protein Rheb with increasing concentrations of PDEδ increased the polarization signal, in agreement with binding of the rotationally highly mobile peptide to the relatively immobile PDEδ protein (Fig. 1A). By contrast, no change in polarization was observed, if the peptide was incubated with purified Gal-1 at concentrations up to 50 μM (Fig. 1B), confirming very recently published results^43^.

Gal-1 specifically recognizes active H-ras and should therefore in addition to the farnesylated C-terminal hypervariable region (hvr) of Ras also recognize its G-domain^35^. We therefore next investigated whether Gal-1 directly binds to this major part of H-ras. Following the N-terminal labelling rational from cellular FRET-experiments^12^, we produced H-ras and Gal-1 proteins with an N-terminal acyl carrier protein (ACP)-tag, A1^44^, that is amenable to specific fluorescent labelling^45^ (Supplementary Fig. 1C). Both the purified His-A1-tagged Gal-1, as well as the final processed A1-tagged Gal-1, retained their lectin-binding ability in a hemagglutination assay (Supplementary Fig. 1D). As expected, we found a significant increase in FRET upon incubation of ATTO-488-labelled GTPγS-H-ras with the DY-547-labelled Ras Binding Domain (hereafter RBD) of the Ras effector C-Raf, which is known to bind to Ras and here served as a positive control. By contrast, incubation with Gal-1 under the same conditions did not show any increase in FRET, as compared to control samples of GDP-H-ras with the C-Raf-RBD or Gal-1, or of fluorophore-only controls (Fig. 1C).

In order to rule out that any missing stable posttranslational modification on either protein or other stable unknown cellular components prevented the GTP-H-ras/Gal-1 interaction, we performed FRET-experiments with A1-tagged purified proteins that were fluorescently labelled and fluorescent-protein tagged proteins obtained from crude mammalian cell lysates. While purified A1-labelled GTPγS-H-ras showed high FRET with lysates from BHK21 cells expressing mRFP-labelled C-Raf-RBD, no FRET was observed with mRFP-Gal-1 lysates (Fig. 1D). Note that the mRFP-tag did not abolish the ability of wt Gal-1 to bind to its ligand lactose (Supplementary Fig. 1E). Binding was only abolished once mutation N47D,W69L^46^,^47^ was in addition introduced, which inactivates the carbohydrate binding site (Supplementary Fig. 1E). In the inverse FRET situation, predominantly GTP-loaded mGFP-H-rasG12V from cell lysates showed significantly increased FRET with DY-547-labeled C-Raf-RBD, while the identically labelled Gal-1 did not show any FRET above background (Fig. 1E). Here, we observed a higher background FRET signal, due to the CoA-547 label, as demonstrated in control experiments (Supplementary Fig. 1F).

In conclusion, these experiments did not support the direct binding of Gal-1 to farnesylated Ras-peptides or active H-ras, thus confirming our initial observations. Hence an alternative model to the one describing a direct, farnesyl-dependent GTP-H-ras/Gal-1 interaction was required.

### Galectin-1 indirectly interacts with active H-ras via the RBD of Ras effectors

**Figure 2:**
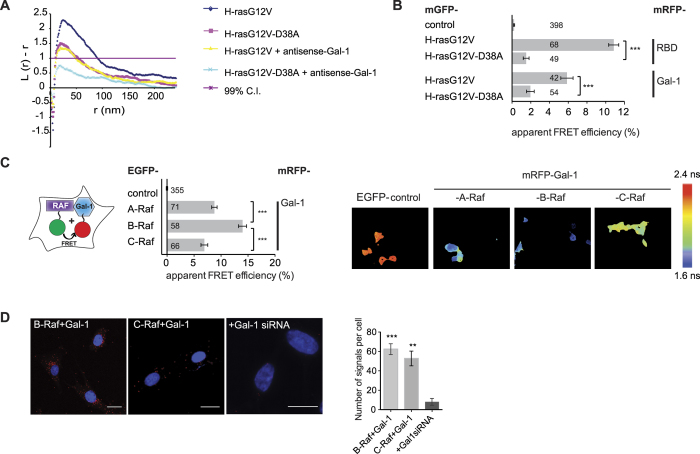
H-rasG12V nanoclustering largely depends on effector interactions, while Gal-1 interacts with Raf-effectors. (**A**) Electron microscopic nanoclustering analysis of mGFP-H-rasG12V and mGFP-H-rasG12V-D38A with or without antisense-mediated knockdown of Gal-1 in BHK21 cells. Normalized univariate K-functions, where maximal *L(r)-r* values above the 99% CI for complete spatial randomness indicate clustering at that value of r (number of membrane sheets analysed per condition, n ≥ 10). (**B**) Complexation between indicated mGFP-tagged H-ras mutants and mRFP-tagged C-Raf-RBD or Gal-1 was determined using FLIM-FRET in HEK293-EBNA cells transiently expressing above constructs (two independent biological repeats). (**C**) Complexation between indicated EGFP-tagged full-length Raf proteins and mRFP-tagged Gal-1 measured by FLIM-FRET in HEK293-EBNA cells (three independent biological repeats). Examples of FLIM-FRET images of cells, coexpressing indicated FRET-pairs or EGFP-tagged C-Raf-RBD as donor-only control. Image colour look-up table on the right shows fluorescence lifetimes. (**B,C**) Plotted values correspond to the mean ± SEM. Numbers inside and above the bars indicate total number of cells imaged. The Methods section describes the indicated statistical comparisons (***p < 0.001). Samples with coexpressed fluorescent proteins mGFP and mRFP (**B**), or EGFP and mRFP (**C**) served as FRET controls. Note that non-control sample FRET-values were all significantly different from the (FRET-)control sample. (**D**) Analysis of the interaction between endogenous Raf isoforms and Gal-1 in BHK21 cells using *in situ* proximity ligation assay (PLA). Representative confocal microscopy images of indicated proteins are shown. The sample with siRNA-mediated Gal-1 depletion served as a negative control. Cell nuclei were stained with DAPI. Red foci indicate positive signals for protein interactions and their quantification is shown in the graph. Scale bar is 21 μm.

When studying nanoclustering of H-rasG12V mutants, we serendipitously found that the effector-site mutation D38A^48^ reduced nanoclustering to a similar extent as knockdown of Gal-1, and a complete loss of nanoclustering was observed when knockdown and mutation were combined (Fig. 2A). We therefore tested, whether residue D38A, which is at the centre of the Ras/RBD interface^48^, affects the FRET between mRFP-Gal-1 and mGFP-H-rasG12V in HEK293-EBNA cells. Indeed, the Ras effector-site mutation did not only abrogate FRET between H-rasG12V and the C-Raf-RBD, but also of H-rasG12V and Gal-1 (Fig. 2B). Note that FRET-levels were significantly lower than those of the non-farnesylatable CAAX-mutant (Supplementary Fig. 1B), supporting that farnesylation was not required for an interaction with either the RBD or Gal-1 in cells, while an intact effector-site on H-ras was required. These data therefore indicated that GTP-H-ras/Gal-1 complex formation depends on binding of an effector to H-ras and may therefore proceed indirectly.

Gal-1 overexpression was previously shown to increase and prolong EGF-stimulated signalling output of Raf, while apparently suppressing that of PI3K, the other major Ras effector^36^. We therefore tested, whether Gal-1 forms FRET-competent complexes with the Raf kinases (Supplementary Fig. 2A), which could bridge the interaction to active Ras. We coexpressed EGFP-tagged Raf paralogs and mRFP-tagged Gal-1 and monitored their interaction in HEK293-EBNA cells grown under normal serum levels using FRET. As compared to the H-rasG12V interaction with Gal-1 (Fig. 2B), all Raf paralogs showed similar FRET with Gal-1, with B-Raf displaying the highest FRET (Fig. 2C). Moreover, Proximity Ligation Assay (PLA) in BHK21 cells confirmed interaction of endogenous Gal-1 with B- and C-Raf (Fig. 2D).

In agreement with a significant requirement of Raf-proteins for mediating Gal-1 induced H-rasG12V-nanoclustering, knockdown of B-Raf abolished the Gal-1 induced increase in FRET (Supplementary Fig. 2B), indicating a significant role of B-Raf in H-rasG12V nanoclustering. Interestingly, knockdown of A-Raf significantly decreased nanoclustering of H-rasG12V in the presence of Gal-1 below control levels (Supplementary Fig. 2B). These data may suggest an important role for A-Raf in independently scaffolding GTP-H-ras nanocluster or preventing a negative effect on these nanocluster in the presence of Gal-1.

**Figure 3:**
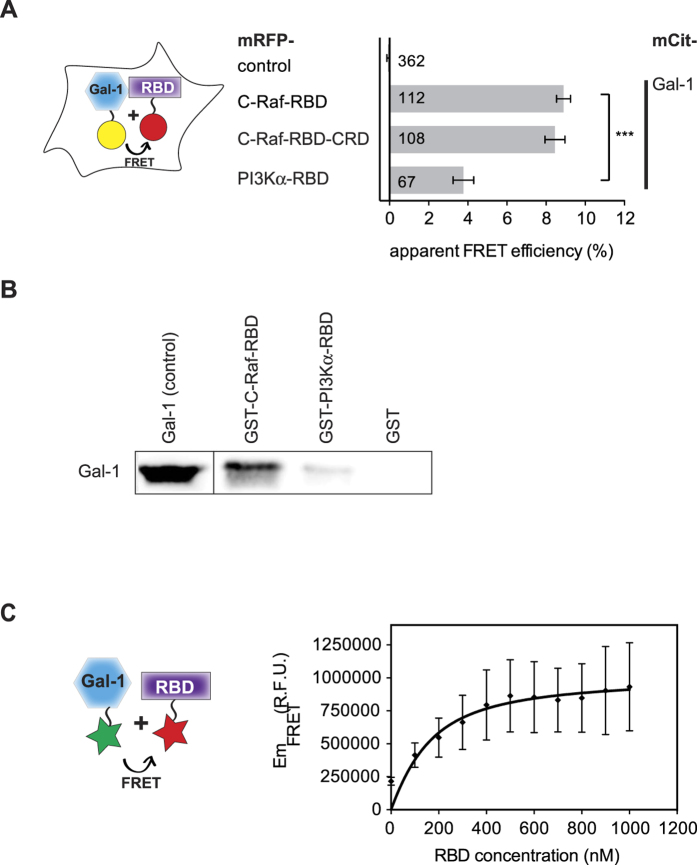
Galectin-1 directly binds to the Ras binding domain of effectors. (**A**) Interaction of Gal-1 with fragments of C-Raf (as can be derived from Supplementary Fig. 2A) or with the RBD of PI3Kα studied by FLIM-FRET in BHK21 cells, transiently expressing mCit-tagged Gal-1 and mRFP-tagged RBD-constructs (three independent biological repeats). Fluorescence lifetimes of FRET-samples were all significantly different from the donor-control. Plotted values correspond to the mean ± SEM. Numbers inside and above the bars indicate total number of cells imaged. The Methods section describes the indicated statistical comparisons (***p < 0.001). Samples with coexpressed fluorescent proteins mRFP and mCit served as FRET controls. Note that non-control sample FRET-values were all significantly different from the (FRET-)control sample. (**B**) GST pull-down experiments were performed by mixing bacterially purified Gal-1 with GST, GST-C-Raf-RBD or GST-PI3Kα-RBD immobilized on glutathione sepharose beads. GST was used as a negative control. Proteins retained on the beads were resolved by SDS-PAGE and Western blotted using a monoclonal antibody (M01) against Gal-1 for detection. (**C**) Corrected sensitized acceptor emission FRET data of 100 nM ATTO-488-labelled Gal-1 titrated with increasing concentrations of DY-547-labelled C-Raf-RBD (scheme on the left). Both proteins were purified from bacteria and labelled with the ACP-tag method. The dissociation constant (K_d_) was determined from the shown curve fit on the dataset of Em_FRET_ as described in the Methods section. Plotted values correspond to the mean ± SEM.

Next, we wanted to identify the minimal domain of Raf that mediates the interaction with Gal-1. The D38A mutation reduces the affinity of GTP-H-ras to the C-Raf-RBD by approximately 100-fold to 1300 nM, thus basically abrogating their interaction^48^. Based on our observations that this mutation also blocks H-rasG12V complexation with Gal-1 (Fig. 2B), we reasoned that RBD containing Raf-fragments would bind to Gal-1. Indeed, both the C-Raf-RBD and the same extended by the cysteine rich domain (CRD) showed identically high FRET with Gal-1 when expressed in BHK21 cells (Fig. 3A). Moreover, the structurally related RBD from PI3Kα showed FRET with Gal-1, albeit significantly less than the C-Raf-RBD (Fig. 3A, Supplementary Fig. 3B), suggesting that Gal-1 directly interacts with the Ras binding domain of effectors. In order to confirm these FRET results, we performed co-immunoprecipitation experiments, which showed that GST-labelled RBD-fragments were able to pull-down bacterially purified Gal-1 (Fig. 3B) or *vice versa* (Supplementary Fig. 2D). Finally, we provided evidence for direct binding of Gal-1 to the RBD. We purified Gal-1 and the C-Raf-RBD, both with N-terminal A1-tags in order to label them fluorescently for FRET-based binding experiments (Fig. 3C). Analysis of our FRET-binding data established a dissociation constant for Gal-1/C-Raf-RBD of K_d_ = 106 ± 40 nM.

In conclusion, our results demonstrate that Gal-1 binds directly and with submicromolar affinity to the Ras-binding domain of effectors. This binding mode finally explains how Gal-1 selectively recognizes active Ras, namely by co-recruitment with effectors to active Ras.

### Loss of galectin-1 binding by mutating D117 in the RBD provides tentative support for a computational model of the galectin-1/RBD complex

**Figure 4:**
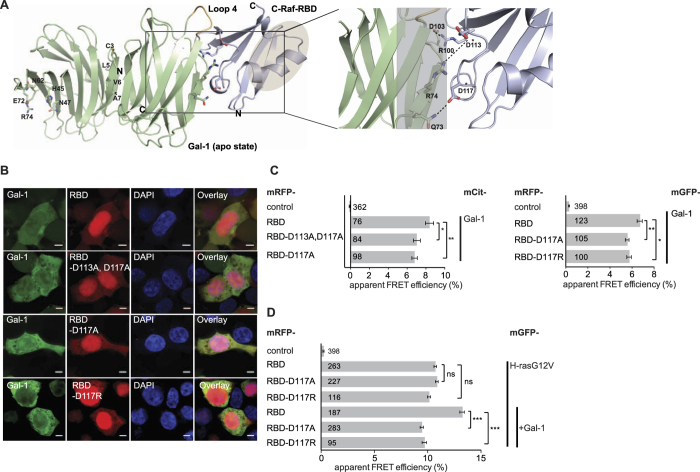
Computational modelling and mutational validation of the Galectin-1/RBD complex. (**A**) Computational representation of a monomer of Gal-1 (*3W58*) and C-Raf-RBD (*1C1Y*: herein RBD)^52^ complex from an optimized low energy molecular docking pose superimposed with dimeric Gal-1 from the same PDB deposition. Numbering of residues is according to sequences deposited in UniProt (P09382 – Gal-1_*Homo sapiens,* P04049 – C-Raf_*Homo sapiens)*. The loop (loop 4) that undergoes major conformational and stereo-chemical changes between apo- and liganded Gal-1 is coloured orange (Supplementary Fig. 3A). *Left:* Note that the Gal-1 dimer interface, marked by the four mutated residues, is to the left near the N-terminus (N) of Gal-1. Residues forming the CBS are shown on the left monomer. The grey oval marks the region on the C-Raf-RBD that contacts Ras. *Enlarged panel to the right* shows a close-up view into the putative protein-protein interface and major interactions. Residues that were mutated in the RBD and showed an effect are marked with asterisks. The uncertainty regarding the interacting surface on Gal-1 is indicated by the translucent grey box. (**B**) Representative confocal images of HEK293-EBNA cells co-transfected with mGFP-Gal-1 and mRFP-C-Raf-RBD (RBD) mutated in the indicated residues. Columns represent imaged fluorescent channels, appropriate for the indicated construct. The nucleus is stained by DAPI. Overlay images show superposition of images to the left. Scale bar is 5 μm. (**C**) Interaction between mCit-Gal-1 (left) or mGFP-Gal-1 (right) and mRFP-tagged C-Raf-RBD and derived interfacial mutants studied using FLIM-FRET in HEK293-EBNA cells transiently expressing indicated constructs (three independent biological experiments). (**D**) Interaction between mGFP-H-rasG12V and mRFP-tagged C-Raf-RBD and derived interfacial mutants with or without coexpressed non-labelled Gal-1 (+Gal-1) studied using FLIM-FRET in HEK293-EBNA cells transiently expressing indicated constructs (three independent biological experiments). (**C,D**) Plotted values correspond to the mean ± SEM. Numbers inside the bars indicate total number of cells imaged. The Methods section describes indicated statistical comparisons (ns, non significant; *p < 0.05; **p < 0.01; ***p < 0.001). Samples with coexpressed fluorescent proteins mCit and mRFP (**C**) or mGFP and mRFP (**C,D**) served as a FRET control. Note that non-control sample FRET-values were all significantly different from the (FRET-)control sample.

In order to have an experimentally testable model of the complex between Gal-1 and the C-Raf-RBD, we conducted computational docking using the existing crystal structural data of Gal-1 and the C-Raf-RBD (Fig. 4A).

The Global RAnge Molecular Matching (GRAMM) methodology, validated on the docking benchmark set and through the Critical Assessment of PRedicted Interactions blind prediction challenge (CAPRI), was employed to build 20 Gal-1 (carbohydrate ligand-bound and -unbound) complexes with the C-Raf-RBD that were ranked highest amongst 1000 docking poses. The software ranks poses by energy minimization and taking the clustering in same local energy minima into account. Highest ranked poses satisfying post-processing experimental constraint filters that are described below, were further refined locally in the RosettaDock web interface for the best fit between the two binding partners.

In order to filter for candidate poses that could represent testable models for the Gal-1/C-Raf-RBD complex, we took three sets of experimental data into account. Firstly, the interface should be common to Gal-1 and galectin-3 (Gal-3; Supplementary Fig. 3B,C), i.e. close to the conserved carbohydrate binding site (CBS), but possibly not identical, as a thiodigalactoside-derived inhibitor of Gal-1 and Gal-3 with submicromolar affinity to Gal-3 (K_d_ = 29 ± 7 nM)^49^ did not affect cellular FRET between Gal-1 and the C-Raf-RBD (Supplementary Fig. 3A). Importantly, this suggests that classical inhibitors of galectins that compete with the β-galactoside-ligand do not interfere with the Gal-1/RBD-interaction. In agreement with this, also H-rasG12V nanoclustering remained unaffected by the compound (Supplementary Fig. 3A). Secondly, Gal-1 interacts with both the C-Raf- and the PI3Kα-RBD (Fig. 3A), suggesting that the interface must span conserved regions of these two proteins (Supplementary Fig. 2C). Thirdly, the Gal-1 interface with the RBDs cannot overlap with their Ras binding region, as Gal-1, RBD and Ras would form concurrently a complex.

An initial complex of the Gal-1 monomer in the ligand bound state (i.e. prepared from dimeric, lactose bound Gal-1, PDB ID: *1GZW*)^50^ and the C-Raf-RBD (PDB ID: *1RFA*)^51^ was made in GRAMM compiler and iteratively refined (Supplementary Fig. 3D). Based on this model we generated Gal-1- and RBD-mutants, in order to experimentally validate the Gal-1/C-Raf-RBD interaction surface (Supplementary Table 1). Three initial mutants of the C-Raf-RBD from the lowest energy poses of Gal-1 with the C-Raf-RBD that did not undergo subsequent refinement in RosettaDock predominantly localized to the nucleus, unlike their wt counterpart (Supplementary Fig. 3E), which obviated further Gal-1 interaction analysis. Sequence analysis revealed that previously unrecognized nuclear localization- (NLS) and nuclear export signals (NES) appear to be localized in the C-terminal part of the RBD of C-Raf (Supplementary Fig. 2C). Two Gal-1 mutants that were generated based on this first, ligand-bound Gal-1 pose (Supplementary Fig. 3D) localized normally, but did not show any loss of function (Supplementary Fig. 3F,G).

Considering that there are no known intracellular carbohydrate ligands, we used a Gal-1 monomer in the ligand unbound (apo-) state prepared from dimeric Gal-1 (PDB ID: *3W58*) docked with the C-Raf-RBD (PDB ID: *1C1Y*)^52^ to build an alternative lowest-energy refined pose (Fig. 4A). Here, residues D113 and D117 formed ionic and hydrogen bonds with Gal-1′s R74 and Q73, respectively. In addition R100 from the RBD formed an ionic bond with Gal-1′s D103. From both structural models (Fig. 4A, Supplementary Fig. 3A), three C-Raf-RBD mutants could thus be derived, RBD-D113A, D117A and RBD-D117A, as well as the charge reversing counterpart RBD-D117R. All of them localized to the cytoplasm and nucleus like the parent, wild type (wt) C-Raf-RBD (Fig. 4B). Analysis of their interaction with Gal-1 in cells using FRET revealed a significant loss of function in their interaction with Gal-1 (Fig. 4C). Importantly, mutation D117A or D117R in the C-Raf-RBD was sufficient to desensitize it for Gal-1 enhanced recruitment to H-rasG12V (Fig. 4D).

The mutation of Arg 100, another residue in the modelled interface (Fig. 4A) to Asp did already affect the RBD-recruitment FRET to H-rasG12V (Supplementary Fig. 3H). This suggested that overall properties of the RBD affinity to Ras were compromised. Indeed, a previous report stated that only if R100 was mutated to Ala, would the affinity of the RBD for H-ras be basically unaffected^53^. Mutational analysis of the Gal-1 interface was inconclusive, as the mutation Q73A did not have an effect (Supplementary Fig. 3G).

In conclusion, a structural docking derived C-Raf-RBD mutant that is deficient in Gal-1 binding is also insensitive to Gal-1-dependent (nanoclustering mediated) enhanced recruitment to active H-ras^10^. These data support the C-Raf-RBD interface of our tentative structural model, while that of Gal-1 remains unclear. Therefore only high-resolution structural approaches (of full length proteins in the context of the membrane) can resolve the actual details of the complex, and we wish to clarify that our model represents merely a proposition of how Gal-1 and the C-Raf-RBD might interact, based on our experimental data.

### The galectin-1 dimer interface is required to modulate positively GTP-H-ras-, and negatively GTP-K-ras-nanoclustering

Inhibitors of Raf that induce its dimerization were recently shown to increase Ras nanoclustering. Likewise an artificial tandem-fusion protein of the B- and C-Raf RBD-CRD was sufficient to stabilize Ras nanoclustering^54^. Given the very similar nanoscale organization of the effector Raf with Ras^9^,^55^, it is plausible to assume that dimeric Gal-1 stabilizes effector dimers to augment nanoclustering.

We therefore tested the hypothesis that an intact Gal-1 dimer-interface is necessary for H-rasG12V-nanoclustering and -signalling promotion. Mutations at the N-terminus of Gal-1 were previously described in two dimerization compromised mutants (Gal-1-C3S,L5Q,V6D,A7S; K_d_ ≈ 250 μM and Gal-1-V6D; K_d_ ≈ 60 μM)^56^. However, the single mutation of residue V6D, did not lead to a loss of dimerization-FRET in HEK293-EBNA cells (Supplementary Fig. 4A). By contrast, the mutant that combined all N-terminal mutations, here named N-Gal-1, showed significantly decreased dimerization-FRET in HEK293-EBNA cells as compared to the non-mutated parent (Supplementary Fig. 4A), as well as reduced amounts of the dimer in a native gel analysis (Supplementary Fig. 4B).

**Figure 5:**
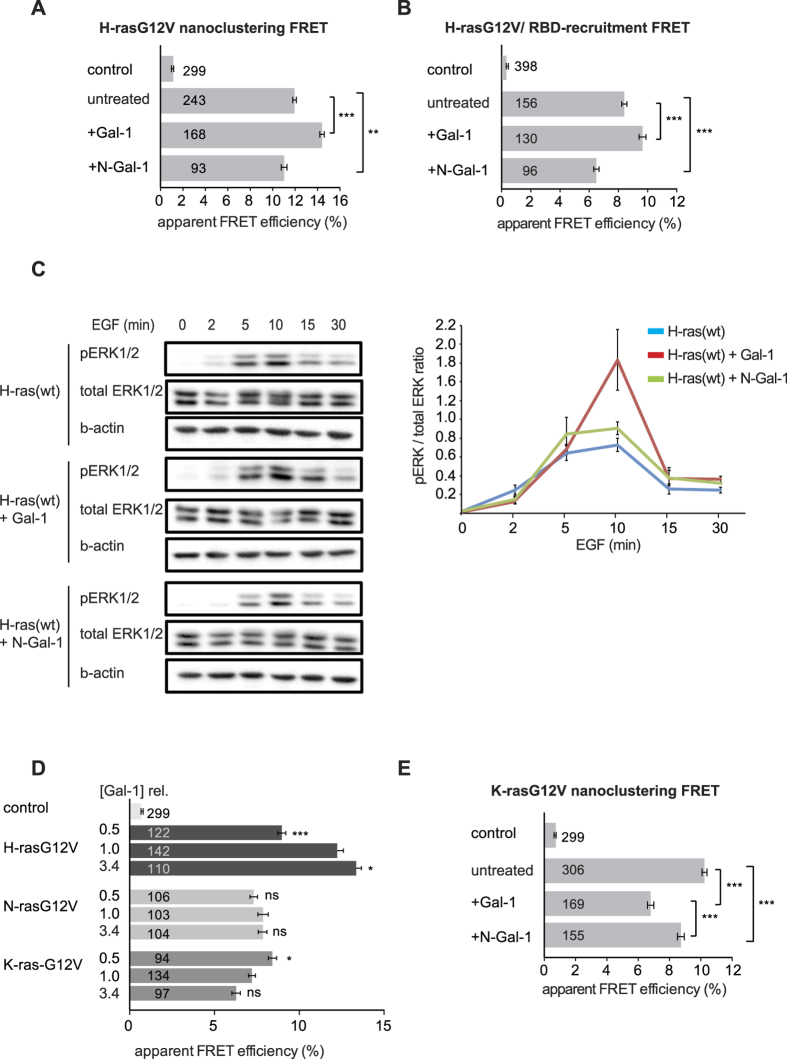
A dimerization deficient Galectin-1 mutant loses its effect on Ras nanoclustering and signalling. (**A**) Nanoclustering-FRET response of H-rasG12V in dependence of Gal-1 or its dimerization deficient mutant N-Gal-1 in HEK293-EBNA cells expressing mGFP-/mCherry-H-rasG12V. (**B**) RBD-recruitment FRET response of H-rasG12V in dependence of Gal-1 or its dimerization deficient mutant N-Gal-1 (mGFP-H-rasG12V and mRFP-C-Raf-RBD expressed in HEK293-EBNA) to assess effector translocation from the cytoplasm to active H-ras in plasma membrane nanoclusters. (**C**) Left, Western blot analysis of HEK293-EBNA lysates expressing mGFP-tagged H-ras and mRFP-tagged Gal-1 constructs as indicated. Serum-starved cells were stimulated with 100 ng/ml EGF for the indicated times. Total ERK and phospho-ERK (pERK) levels were then determined by immunoblotting. β-actin is the loading control. Right, Quantification of three independent repeats of Western blot data as shown on left. The pERK-signal was normalized to the total ERK-signal. (**D**) The nanoclustering-FRET response of H-rasG12V, N-rasG12V and K-rasG12V with increasing concentration of Gal-1. BHK21 cells were transiently co-transfected with mGFP-/and mCherry-tagged Ras constructs alone (1.0) or with antisense-Gal-1 (0.5) or non-labelled Gal-1 (3.4). The cellular total Gal-1 concentration relative to endogenous Gal-1 in control BHK21 cells ([Gal-1]rel.) is displayed to the left of the data. (**E**) The nanoclustering-FRET response of K-rasG12V. HEK293-EBNA cells transiently expressed mGFP-/mCherry-K-rasG12V and if indicated non-labelled Gal-1 or N-Gal-1. Note that in (**D**) the FRET-levels for K-rasG12V are lower than in (**E**), due to the higher Gal-1 level in BHK21 as compared to HEK293-EBNA cells (Supplementary Fig. 4E). (**A,B,D,E**) Plotted values correspond to the mean ± SEM of three independent biological experiments. Numbers inside the bars indicate total number of cells imaged. The Methods section describes the indicated statistical comparisons (ns, non significant; *p < 0.05; ***p < 0.001); comparisons in (**D**) were done against the 1.0 parent-control. Samples with coexpressed fluorescent proteins mGFP and mCherry (**A,E**) or mGFP and mRFP (**B**) served as a FRET control. Note that non-control sample FRET-values were all significantly different from the (FRET-)control sample.

While wt Gal-1 significantly increased H-rasG12V nanoclustering (Fig. 5A) and RBD-effector recruitment to H-rasG12V (Fig. 5B) as observed before^10^, N-Gal-1 did not support either increase (Fig. 5A,B). Of note, FRET between the C-Raf-RBD and N-Gal-1 was significantly increased in cells (Supplementary Fig. 4C). This observation rules out that a loss in affinity for the C-Raf-RBD is responsible for the loss in RBD recruitment. Consistent with this loss-of-function in supporting H-rasG12V nanoclustering and RBD recruitment, N-Gal-1 did not potentiate EGF-induced ppERK-signalling when H-ras was overexpressed (Fig. 5C). Hence, our data show that an intact Gal-1 dimer interface is required for H-ras nanocluster augmentation.

In addition to H-rasG12V, K-rasG12V was also originally described to co-immunoprecipitate with Gal-1, opening up the possibility that K-ras nanoclustering could also be affected by Gal-1^36^. Based on FRET-experiments, we could confirm that like H-rasG12V (Supplementary Fig. 1A) both K- and N-rasG12V are in close, FRET-competent complexes with Gal-1 in BHK21 cells, even if the cells were treated with compactin to block farnesylation (Supplementary Fig. 4D). However, when we studied the effect of Gal-1 on Ras nanoclustering in BHK21 cells, we observed that increasing concentrations of Gal-1 had no effect on N-rasG12V, while they negatively regulated K-rasG12V nanoclustering-FRET (Fig. 5D). Consistent with a dimerization dependent negative activity of Gal-1 on K-rasG12V nanoclustering, N-Gal-1 was also less efficient in reducing K-rasG12V nanoclustering than wt Gal-1 (Fig. 5E).

In conclusion, efficient Gal-1 dimerization is required to positively regulate H-rasG12V nanocluster and negatively regulate K-rasG12V nanocluster.

## Discussion

**Figure 6:**
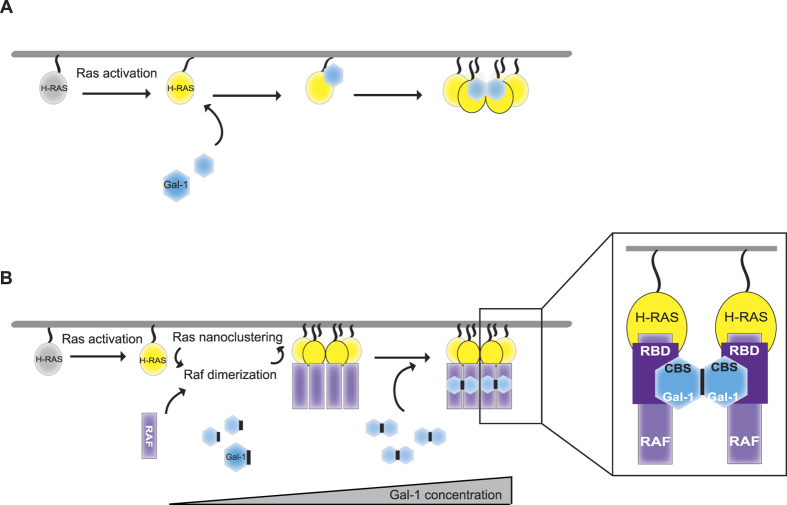
Comparison of current and our new model for the mechanism of action of Gal-1 as a nanocluster scaffold. (**A**) Current model. Direct interaction of active H-ras and Gal-1 stabilizes nanocluster. H-ras (depicted as yellow oval) activation supposedly makes the C-terminal farnesyl chain of H-ras more accessible for the prenyl-binding pocket of Gal-1 (depicted as a blue hexagon). This mechanistic step has not been described for other, similar trafficking chaperones, such as GDIs or PDEδ. Instead the spontaneous, activation state independent dissociation from the membrane is the basis for complexation by such chaperones in the cytoplasm^74^,^75^. (**B**) In the new model proposed here, Raf effectors (depicted as violet rectangles) are recruited to active H-ras in nanoclusters on the plasma membrane. At higher concentrations Gal-1 can dimerize. Gal-1 binds directly to the Ras binding domain (RBD) of effectors, such as Raf. Thus, dimeric Gal-1 could stabilize effector (Raf)-dimers, which then act as the actual ‘scaffold’ for H-ras nanocluster. Note that effector and Gal-1 can form complexes already in the cytoplasm. Stacked dimers of H-ras + effector (Raf) + Gal-1 (box to right) would be nucleating the growth of H-ras nanocluster, a process that may be supported by the membrane environment.

Ras nanoclustering is indispensable for Ras signalling^8^ and we have recently described it as target of cancer associated Ras-mutations^57^, underscoring its significance for the signalling architecture of Ras. Only a handful of nanocluster regulators, so called Ras nanocluster scaffolds are known, and Gal-1 has so far been the one scaffold that was functionally and mechanistically best understood. We here presented data, which question the existing model of Gal-1 binding directly to the farnesyl-lipid on the C-terminus of Ras proteins (Fig. 6A). We did neither observe binding of Gal-1 to a farnesylated Ras-peptide, nor directly to the G-domain of Ras (Fig. 1). Instead, we found that Gal-1 indirectly couples to Ras via a direct association with the RBD-domain of effectors (Figs 2, 3, 4) and that an intact Gal-1 dimer interface is required for Gal-1 to modulate Ras nanoclustering (Fig. 5).

Others previously suggested binding of farnesylated proteins to Gal-1. Two different mutations were described that abrogated binding to farnesyl, K28T (according to our numbering K29T)^41^ and L11A (L12A, likewise)^33^. These mutants were brought in agreement by proposing a farnesyl-binding pocket along the N-terminal or dimer interface part of Gal-1^41^. However, we did not observe any effect of the former mutation on the complexation of Gal-1 and H-rasG12V (Supplementary Fig. 1B). It is conceivable that the L11A mutation near the dimer interface of Gal-1 affects the ability of Gal-1 to dimerize and thus H-ras-GTP nanoclustering. However, this has not been shown so far.

With our new model (Fig. 6B), we resolve inconsistencies of the previous model, such as how specificity for active Ras is mediated and incorporate recent findings, which demonstrated that Raf dimer-inducing compounds do also increase Ras nanoclustering^54^. Thus we propose the following revised mechanistic model for the function of Gal-1 as a nanocluster scaffold (Fig. 6B): upon Ras activation and recruitment of the effectors to Ras, Gal-1 binds with high affinity to the accessible part of the RBD of effectors. Note that according to our data with non-farnesylated H-rasG12V (Supplementary Fig. 1A,B), it is possible that Gal-1 and effectors directly bind to each other in the cytoplasm. As Gal-1 can dimerize at μM concentrations that can be found in mammalian cells^10^, it could stabilize effector dimers, such as e.g. Raf-dimers. We therefore here propose that the Raf-dimers are the actual nanocluster stabilizer. This is supported by our data showing that loss of the effector binding capability of H-rasG12V-D38A (Fig. 2B) and knockdown of A- and B-Raf (Supplementary Fig. 2A) can dramatically reduce Gal-1 supported H-rasG12V nanoclustering. This model is furthermore consistent with the activity of artificially fused dimeric RBD-CRD to stabilize nanocluster^54^. Our model is also in agreement with data that revealed a clustered organisation of Raf on the membrane^18^,^55^. Thus the idea is corroborated that Ras-nanoclusters represent dynamic signalling hubs of Ras and its effectors.

Our new model brings about a ‘chicken-and-egg’ problem, namely whether nanoclustering enhances effector recruitment^10^ or effector binding to Ras enhances nanoclustering. Given the dense ‘lattice’ of nanoclustered Ras, effectors and scaffolds on the inner side of the plasma membrane, allosteric or configurational cooperativity may at least transiently emerge in Ras nanoclusters, which could resolve this apparent paradox. Effector dimerization most likely leads to the cooperative growth of Ras nanocluster, which conversely increases the recruitment probability of more effectors. The activity of this system would be strictly limited by the highly dynamic nature of the Ras-effector interaction (high off-rate of effectors) and ultimately by the GTP/GDP-exchange cycle of Ras.

This model may also explain the observation that Gal-1 apparently shifts the H-ras activity from the PI3K to the Raf pathway. The higher effective affinity (i.e. as judged by our cellular FRET-experiments) of Gal-1 for the RBD of C-Raf *vs.* PI3Kα (Fig. 3A) could explain, how Gal-1 shifts the signalling output relatively from PI3K to Raf, an effect that could be potentiated in a nanocluster.

However, our model still cannot explain how selectivity for H-ras is realized. We propose that the H-ras-effector-Gal-1 complex is conformationally favoured, given that H-, N- and K-ras exhibit apparently distinct reorientation properties on the plasma membrane, with GTP-H-ras contacting the membrane via its helix α4^12^,^58^. Likewise, certain effectors or effector paralogs might be favoured by different conformational mechanisms. From our data one might speculate that A- and B-Raf are particularly relevant for H-ras nanoclustering, a constellation, which would be somewhat complementary to the apparent preference of N- and K-ras nanocluster for B- and C-Raf^54^.

How does Gal-1 negatively impact on K-ras nanoclustering? It is important to note that the dimer-interface mutant N-Gal-1 does not lead to an increase of K-ras nanoclustering as compared to Gal-1 depletion (compare Fig. 5D,E). This means that the N-Gal-1 mutant is just less potent than the wt Gal-1 to affect K-ras nanoclustering, while another component upstream may actually mediate the effect on K-ras. We therefore propose that the effect of Gal-1 on K-ras nanoclustering depends on GTP-H-ras, which was recently shown to negatively regulate K-ras nanoclustering by redistributing phosphatidyl-serine in the plasma membrane^17^. Thus GTP-H-ras nanocluster would negatively regulate K-ras, an intriguing antagonistic constellation of these two Ras isoforms, which might be associated with the very different mutation rates of these elemental switches in cancer.

Currently a number of inhibitors for galectins are being developed^29^,^32^,^59^. However, their intended mechanism of action is exclusively focused on the functions of galectins in the extracellular space. Most of these compounds are β-galactoside analogs that are often not cell permeable and would compete with natural ligands of galectins in the extracellular space. Our current data show that a cell-permeable inhibitor of that type does not interfere with the binding of Gal-1 to the C-Raf-RBD, opening up the possibility of a distinctly targetable interface. Another interesting aspect is that this new interaction site would overlap with the NWGR anti-death motif in Gal-3^60^. However, we have not investigated Gal-3 further in the current study.

Finally, our data suggest that Salirasib may have a different molecular target than Gal-1 to exert its anti-cancer activity (e.g. PDEδ could be a candidate). Given Salirasib’s success to progress in clinical trials, it may be crucial to establish its true target in order to support the development of similar therapeutic approaches in the future.

## Methods

### Plasmids and molecular cloning

Different expression plasmids for *in vitro* and cellular experiments were used. We obtained plasmid pEGFP-C-Raf as a kind gift from Prof. Krishnaraj Rajalingam, pEGFP-A-Raf from Dr. Angela Baljuls and pEGFP-B-Raf from Prof. John F Hancock.

Plasmids pmRFP-Gal-1, pmRFP-C-Raf-RBD, pmRFP-PI3Kα-RBD, pmRFP-C-Raf-RBD-CRD and pmGFP-H-rasG12V were described in ref. 12,58, while plasmids pcDNA3-antisense-Gal-1 and pcDNA3-Gal-1 in ref. 35. Plasmids pQE-A1, pQE-A1-H-ras(wt) and pQE-A1-C-Raf-RBD were described in ref. 10. Plasmids pmGFP-H-ras(wt), pmCherry-H-rasG12V, pmGFP-K-rasG12V, pmCherry-K-rasG12V, pmGFP-N-rasG12V and pmCherry-N-rasG12V were described elsewhere^57^. Plasmid pmGFP-H-rasG12V-D38A was prepared by mutating the H-rasG12V parent. pmCit-Gal-3, pmCit-Gal-1 were generated by cloning galectins from the mRFP-vectors into the pmCit-C1 vector. To generate construct pQE-A1-Gal-1, sequence of Gal-1 was first amplified from plasmid pmRFP-Gal-1 by PCR using forward primer 5′-TCAGATCTCATGGCCTGTGGTCTGGTC- 3′ and reverse primer 5′- GAGGTACCTTTATCACTCAAAGGCCACACAC -3′ (Sigma-Aldrich). Amplified PCR product was purified and sub-cloned into pCR™ II-Blunt-TOPO (Invitrogen). From there, the Gal-1 sequence was sub-cloned into BglII and KpnI restriction sites of the pQE-A1 vector. Plasmid pmRFP-C-Raf-RBD served as a template to which mutations were introduced by site-directed mutagenesis (GenScript USA Inc.) in order to generate plasmids pmRFP-C-Raf-RBD-W114,T116A,L121A (C3A); pmRFP-C-Raf-RBD-K109A,W114A,T116A (N3A); pmRFP-C-Raf-RBD- K109A,W114A,T116A,L121A,E124A,L126A (6A); pmRFP-C-Raf-RBD-D117A, pmRFP-C-Raf-RBD-D113A,D117A, pmRFP-C-Raf-RBD-D117R and pmRFP-C-Raf-RBD-R100D. Gal-1 mutants pmRFP-Gal-1-S63A; pmRFP-Gal-1-S63A,D65A; pmRFP-Gal-1-C3S,L5Q,V6D,A7S (pmRFP-N-Gal-1), pmRFP-Gal-1-V6D, pmRFP-Gal-1-K28T and pmRFP-Gal-1-N47D,W69L were constructed by site-directed mutagenesis on the plasmid pmRFP-Gal-1 (GenScript USA Inc.). pmGFP-H-rasG12V-C186S was generated on the plasmid pmGFP-H-rasG12V (GenScript USA Inc.). To generate plasmid pcDNA3-N-Gal-1, the N-Gal-1 sequence from pmRFP-N-Gal-1 was sub-cloned into the HindIII and BamHI sites of the plasmid pcDNA3-Gal-1. All constructs were verified by sequencing (GATC Biotech and GenScript USA Inc.).

### Protein preparation and protein fluorescent labelling for *in vitro* studies

A1-tagged proteins H-ras, Gal-1 and C-Raf-RBD proteins were expressed in *Escherichia coli* using the pQE-expression system (Qiagen, Hilden, Germany) and purified as described^10^. The Ras-binding domains (RBDs) of C-Raf (aa 51–131) and p110α (aa 127–314), the catalytic subunit of PI3Kα, and full-length Gal-1 were produced as glutathione S-transferase (GST) fusion proteins in *E. coli*. The proteins were purified and the GST-tag was cleaved using TEV protease as described before^61^. All proteins were purified and nucleotide exchange for GDP or GTPγS on the H-ras molecule was done as described previously^10^.

The labelling of A1-tagged purified proteins (H-ras, Gal-1 and RBD) was conducted with photostable fluorescent substrates, derivatives of coenzyme A (CoA) (NEB). CoA substrates are based on the ATTO-TEC dye ATTO-488 (CoA-488) or Dyomics dye DY-547 (CoA547) correspondingly and used to label ACP or A1-tagged fusion proteins. The labelling of the fusion proteins with the CoA substrates was performed according to the labelling procedure of the commercial protocol using ACP synthase (NEB, P9301S) from New England Biolabs (NEB). The unreacted substrate was removed by 5 times wash using Amicon^®^ Ultra-2 filters (Millipore). The fluorescent labelling was quantified with Nano Drop 2000c (Thermo Scientific).

### Fluorescence anisotropy

The proteins PDEδ and Gal-1 were expressed in *E. coli* and purified as described^10^,^62^. Rheb peptide (purity > 90%) was purchased from JPT GmbH (Germany). Fluorescence polarization measurements were carried out using a Fluoromax-4 spectrophotometer (HORIBA Jobin Yvon, Munich, Germany). All measurements were carried out at 20 °C in buffer containing 30 mM Tris, 150 mM NaCl and 3 mM Dithiothreitol (DTT) at 495 nm excitation and 520 nM emission wavelengths. Data were analysed using Grafit 5.0 (Erithacus Software, East Grinstead, UK).

### Preparation of cell lysates for protein interaction measurement by FLIM-FRET

BHK21 cells were transiently transfected with the respective plasmids (pmGFP-H-rasG12V, pmRFP-C-Raf-RBD or pmRFP-Gal-1 constructs using JetPRIME transfection reagent (Polyplus-transfection). 24 h after transfection cells were lysed according to the procedure described by Dimauro *et al.* with some modifications^63^. In brief, 4 × 10^5^ cells were washed with phosphate buffer saline and harvested with lysis buffer (50 mM Tris-HCl, 250 mM sucrose, 5 mM MgCl_2_, 3 mM DTT, EDTA-free inhibitor cocktail), incubated on ice for 30 min upon sonication and vortexed for 15 s. The cell debris was pelleted by centrifugation at 800 g for 10 min and total protein amount was measured using the Bradford method (Bio-Rad). The precleared lysates containing overexpressed mGFP-H-ras were mixed with either fluorescently labelled protein DY-547-Gal-1 or DY-547-RBD of final concentration of 1 μM for the latters. The FRET-pair combinations of free fluorescent labels or fluorescent proteins at concentration of 1 μM were used as controls. The supernatants containing overexpressed mRFP-Gal-1 or mRFP-RBD were mixed with H-ras labelled with ATTO-488 and freshly loaded with GTPγS at the final concentration of 1 μM. The final protein/cell lysate mixtures were incubated for 30 min at 4 °C and the samples were measured by FLIM-FRET.

### GST pull-down assay for protein interaction studies

GST pull-down experiments were conducted by adding 30 μM Gal-1 to 30 μM of the GST-fused RBDs of C-Raf and p110α (the catalytic subunit of PI3Kα) immobilized on 30 μl glutathione-conjugated Sepharose 4B beads (Macherey-Nagel, Duren, Germany). The mixture was incubated at 4 °C for 45 min in buffer, containing 30 mM Tris-HCl, pH 7.5, 150 mM NaCl, 3 mM DTT. After washing for three times with the same buffer proteins retained on the beads were resolved by SDS-PAGE and processed for immunoblotting using a monoclonal antibody (M01) against Gal-1 (Abnova). GST was used as a negative control.

### FRET assay *in vitro*

An *in vitro* FRET assay was used to measure FRET between GDP- or GTPγS-loaded H-ras and C-Raf-RBD or Gal-1 in solution. Equal amount of GTPγS-loaded/GDP and ATTO-488-labeled 250 nM H-ras (donors) and 250 nM Dy-547-labeled Gal-1 or RBD (acceptors) as a positive control were used. Measurements were conducted at room temperature in the buffer containing 25 mM Hepes, 100 mM NaCl, 3 mM DTT and 3 mM MgCl_2,_ pH 7.2. Fluorescence spectra were collected from 550 nm to 640 nm after excitation at 470 nm using a Synergy H1 hybrid fluorescence plate-reader (BioTek).

Efficiency of FRET was calculated by measuring the sensitized emission of the acceptor according to


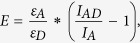


where *ε*_*A*_ and *ε*_*D*_ are the molar extinction coefficients of the acceptor and donor, respectively, at the wavelength of excitation, I_AD_ is the emission of the acceptor in the presence of the donor and I_A_ is the fluorescence of the acceptor-only sample.

FRET assay *in vitro* was also used to determine the affinity between Gal-1 and RBD in solution. Normally, 100 nM of purified and ATTO-488 labeled Gal-1 was titrated with increasing concentrations (100 nM to 1000 nM) of purified and labelled DY-547-labelled C-Raf-RBD in 25 mM HEPES, 100 mM NaCl, 3 mM MgCl_2_, 3 mM DTT pH 7.2 buffer. FRET measurements were performed in a 96-well plate (Perkin-Elmer) with a Synergy H1 hybrid fluorescence plate reader (BioTek, Winooski, VT). Two excitation wavelengths were used: 470 nm to excite ATTO-488-Gal-1 (donor), and 550 nm to excite DY-547-RBD (acceptor). Free dyes ATTO-488 and DY-547 were used as negative control. FRET was measured by sensitized emission of the acceptor using fluorescence spectra collected from 550 nm to 640 nm after excitation at 470 nm. FRET emission of DY-547-RBD (Em_FRET_) was calculated from the total emission of the acceptor in presence of donor by subtraction of the direct emission of donor (donor only) and direct emission of acceptor (acceptor only) according to Song *et al.*^64^. Data processing was done using Gen5 software (version 2.01, BioTek), and K_d_ values were determined as described^64^ using GraphPad Prism 6 software (GraphPad Software Inc., La Jolla, CA). The final Em FRET values were corrected by subtracting background FRET of the free fluorescent dyes.

### Cellular FLIM-FRET analysis

BHK21 or HEK293-EBNA cells were transfected with indicated mGFP- or mCit- tagged donor construct and mCherry- or mRFP-tagged acceptor construct using JetPRIME transfection reagent (Polyplus transfection). For the inhibitor studies cells were treated for 2 h with either control (DMSO 0.2% (v/v)) or 20 μM Di-(3-deoxy-3-(4-((butylamino)carbonyl)-1H-1,2,3- triazol-1-yl)-β-D-galactopyranosyl)sulfane, IC-26-147-1 (batch PRV0312, Galecto Biotech)^49^. DMSO concentration in the final samples was under 0.2% (v/v). Co-transfection with pcDNA3-Gal-1, pcDNA3-N-Gal-1, pcDNA3-Gal-1-V6D or pcDNA3-antisense-Gal-1 was done in order to overexpress Gal-1, N-Gal-1 or Gal-1-V6D or to knockdown Gal-1. Cells were fixed with 4% PFA and mounted with Mowiol 4-88 (Sigma-Aldrich). To knock-down specific Raf paralogs, transfection with 50 nM A-Raf (L 003563-00), B-Raf (L003460-00), C-Raf (L 003601-00) siRNA or scrambled control (D 00181001-05, Dharmacon, GE Healthcare) using JetPRIME transfection reagent was done. Cells were fixed after 48 h. Fluorescence lifetimes of mGFP/mCit (intact cells) or ATTO-488 (cell lysates) were measured using a fluorescence lifetime imaging attachment (Lambert Instruments) on an inverted microscope (Zeiss AXIO Observer D1) as described in ref. 10. Fluorescein (0.01 mM, pH 9) was used as a lifetime reference. Three biological repeats were performed and averages of fluorescence lifetimes from at least 21 cells were calculated. The apparent FRET efficiency was calculated from obtained fluorescence lifetimes^10^.

### Cell culture and confocal imaging

Baby Hamster Kidney (BHK) 21 and Human Embryonic Kidney (HEK) 293-EBNA cells were obtained from ATCC repository. They were grown in Dulbecco’s modified Eagle medium (DMEM) supplemented with 10% FBS, L-glutamine, penicillin (100 U/ml) and streptomycin (100 μg/mL). Both cell lines were grown to a confluency of 80% (8 × 10^7^ cells/ml) and then sub-cultured every 2–3 days.

Subcellular distribution of Gal-1 and RBD mutants was imaged using confocal microscopy. HEK293-EBNA cells, coexpressing mRFP-tagged RBD or Gal-1 mutant with mGFP-tagged wild-type Gal-1 or RBD respectively, were imaged using Zeiss LSM 780 (63×, NA 1.2 water immersion objective, mGFP excitation at 488 nm and mCherry excitation at 543 nm).

### Western blot analysis of ERK activation

HEK293-EBNA cells were transfected with pmGFP-H-ras(wt) alone or co-transfected with pmRFP-Gal-1 or pmRFP-N-Gal-1 using JetPRIME transfection reagent (Polyplus transfection). After 24 h cells, which were previously serum starved for 5 h, were stimulated with 100 ng/ml EGF (Sigma-Aldrich) for 0 min, 2 min, 5 min, 10 min, 15 min and 30 min and lysed using SDS lysis buffer. Cell lysates were separated by SDS-PAGE and blotted using pERK (Cell Signalling, #9106), total ERK (Cell Signalling, #9102) and β-actin (Sigma Aldrich, A1978) antibodies. The Chemidoc MP system (Bio-Rad Laboratories) was used to detect the band intensities, which were then quantified by densitometry in ImageJ software. Band intensities of pERK were normalized to the ones of total ERK. Averages from three different biological repeats were calculated.

### Electron microscopic Ras-nanoclustering analysis

Apical plasma membrane sheets were prepared from BHK cells transiently expressing mGFP-H-rasG12V or mGFP-H-rasG12V-D38A alone or with antisense-Gal-1. mGFP in the plasma membrane sheets was then labelled with 4.5 nm (diameter) gold nano-particles coupled to anti-GFP antibody and digital images were taken at 100,000 × magnification in an electron microscope (Jeol 1011). From the obtained images spatial mapping of the gold particles was performed as described previously^65^. For each sample at least 10 plasma membrane sheets were imaged and analysed. The statistical significance of differences was determined between replicated point patterns using bootstrap test as described in ref. 66.

### Computational modelling and mutational validation

#### Protein Structure and Sequence search

Full length and domain-specific amino acid sequence queries were retrieved from UniProt (http://www.uniprot.org/). X-ray crystal structures for human Gal-1 (*1GZW*, *3W58*), C-Raf-RBD (*1C1Y*) and NMR structure of C-Raf-RBD (*1RFA*) were collected from the RCSB Protein Data Bank. The Invitrogen Vector NTI AlignX module was used for amino acid alignment of human Gal-1 with Gal-3 and of RAS-binding domains (RBDs) of Raf kinases and PI3Kα. Automatic alignments were critically analysed and compared with structure-based alignments in PyMOL.

#### Computational analysis for prediction of NLS and NES stretches

Here we identified nuclear localization signals from target sequences using cNLS mapper predictor model ensuring incorporation of non-canonical NLSs with activity score threshold of 3^67^. Further characterization of the sequences involved prediction of export signals on machine learned NetNES 1.1 server (http://www.cbs.dtu.dk/services/NetNES/). Both approaches (artificial neural networks and hidden Markov models) displayed on the signal plot from which the NES score is calculated were closely inspected for possible mispredictions. The use of point mutations disrupting nuclear exclusion signals and fluorescent cell imaging further confirmed this experimentally.

#### In silico approach to locate protein binding interfaces (PBIs)

For the initial Gal-1/C-Raf-RBD model, global search of the best rigid body conformations was performed according to high-resolution generic docking parameters (Geometric docking^68^). The first stage in GRAMM-X docking starts with searching shape complementarity between the two proteins. This involves simplifying the protein structure as a rigid body representation on a 3D Cartesian grid then searching for degrees of overlap between the pairs of grids with a Fast Fourier Transform (FFT) approach to perform the docking. Next a softened Lennard-Jones potential function is employed to model conformational changes that take place during protein-protein binding^69^. To select for the best conformation from generated hundred to a thousand complexes GRAMM-X re-ranks poses by local minimization with soft van der Waals interaction, clustering of predictions within the same local minima, and rescoring with the target function combining Lennard-Jones^70^ in the second stage of docking. No refinement that allows side-chain or backbone flexibility is available during the GRAMM-X docking steps. GRAMM-X displays final top scoring models based on soft Lennard-Jones potential, evolutionary conservation of predicted interface, statistical residue-residue preference, volume of the minimum, empirical binding free energy and atomic contact energy^69^,^70^. The web based GRAMM-X software (http://vakser.compbio.ku.edu/resources/gramm/grammx/) with default parameters was used for the optimized model. Both low-energy (high scoring) models have been refined locally in RosettaDock server^71^. Output PDB entries were further analysed for interface complementarity, area, and residue interactions using the PISA server (http://www.ebi.ac.uk/pdbe/prot_int/pistart.html) or manually in Discovery Studio (version 4.0, www.accelrys.com).

### *In Situ* Proximity Ligation Assay (PLA)

BHK21 cells cultured on coverslips were transfected with scrambled control (D 00181001-05, Dharmacon, GE Healthcare) or siRNAs targeting Gal-1 (FlexiTube, GS3956, Qiagen) at a final concentration of 50 nM. After 48 h cells were serum starved for 5 h and then stimulated with 100 ng/ml EGF (Sigma-Aldrich) for 10 min before they were fixed with ice-cold methanol. Samples were incubated with mouse monoclonal anti C-Raf (H-8, lot #H0712, Santa Cruz Biotechnology) or anti B-Raf (F-7, lot #B271, Santa Cruz Biotechnology) antibodies and rabbit polyclonal anti Gal-1 antibody (PeproTech USA) for 120 min. Proximity ligation was carried out with the Duolink II *in situ* PLA kit (Sigma Aldrich) according to the instructions of the manufacturer (Olink Biosciences, Sweden). Coverslips were mounted on glass slides using Duolink^®^ *in situ* mounting medium containing DAPI (Sigma Aldrich). At least three confocal images of each sample were acquired using a Zeiss LSM 780 (63×, NA 1.2 water immersion objective, DAPI excitation at 360 nm, detection 461 nm and Duolink^®^ *in situ* detection reagent Red excitation at 594 nm, detection 624 nm). The protein interaction signal per cell was analysed in ImageJ software. In brief, the total number of PLA signal foci were counted and normalized to the number of cells (identified by DAPI stained nuclei).

### Lactose-binding activity of mRFP-Gal-1 (Lactose agarose affinity assay)

HEK293-EBNA cells transiently expressing mRFP-Gal-1(wt) and mRFP-Gal-1-N47D,W69L^46^,^47^ constructs were harvested with lysis buffer (25 mM HEPES, 100 mM NaCl, 5 mM EDTA, 0.5% Triton, pH 7.5) without DTT and immobilized on lactose agarose beads (Vector Laboratories, Inc.) for 1 h following the equilibration, binding, and washing steps in the protocol for immunoprecipitation of GFP-fusion proteins using GFP-Trap^®^_A (ChromoTek GmbH, Germany). 250 mM lactose (10 mM HEPES, 150 mM NaCl, pH 7.5) was used to displace the retained fraction of Gal-1. Samples were resolved by SDS-PAGE and blotted with human Gal-1 antibody (PeproTech USA).

### Lectin activity of purified Gal-1 variants (Hemagglutination assay)

Mouse red blood cells were prepared and suspended in PBS buffer according to a published assay protocol (Guillaume, St-Pierre, Valérie, M., Sachiko, S., Purification of recombinant human galectin-1(Gal-1). Available at: http://jcggdb.jp/GlycoPOD (2014)). Purified A1-Gal-1 and His-A1-Gal-1 (Supplementary Fig. 1C) were quality checked for any sign of degradation on Coomassie stained SDS-PAGE gels. Both proteins were serially diluted (1:2) into a V-shaped 96-well microtiter plate in the buffer containing 25 mM HEPES, 100 mM NaCl, 2 mM DTT and 3 mM MgCl_2_, pH 7.2, starting from 10 mM Gal-1 concentration. An increasing gradient (20–45 μl) of the red blood cell suspension is then added to the wells to evaluate the optimal volume and avoid false positives. Similarly diluted (1:2 dilution with 10 mM starting concentration) concanavalin A was a positive control in this experiment, while equivalent volume of buffer without a lectin acted as a negative control. Instead of presenting the observed formation of spread out agglutinated red blood cells (positive result) versus a clearly defined sediment of non-agglutinated cells (negative outcome), we quantitated the agglutination effect using spectral absorbance^72^ between 400–800 nm after 24 h incubation at 4 °C. The mean maximum OD value from two peaks in that wavelength range was recorded with a Synergy H1 hybrid fluorescence plate reader (BioTek) to determine and compare the agglutination capacity of His-A1-Gal-1 and A1-Gal-1 against concanavalin A mean maximum OD values. Overlapping OD values at desired concentration ranges showing galectin-induced versus concanavalin A hemagglutination were displayed for 30 μl red blood cell suspension.

### Galectin-1 dimer/monomer equilibrium detection by native PAGE

HEK293-EBNA cells transiently expressing untagged Gal-1, mRFP-Gal-1(wt) and mRFP-N-Gal-1 constructs were harvested with lysis buffer (25 mM HEPES, 100 mM NaCl, 5 mM EDTA, 0.5% Triton, pH 7.5) without DTT and were resolved by native PAGE as described in ref. 73. In brief, stacking and resolving gels were prepared using 1.5 M Tris- buffer without SDS. For running/electrode buffer 100 mM Tris-tricine without SDS was used. Membranes were blotted with human Gal-1 antibody (PeproTech USA).

### Statistical analysis

For experimental data statistical differences were determined using an analysis of variance (one-way ANOVA) complemented by Tukey’s honest significant difference test (Tukey’s HSD). The software GraphPad Prism 6 (GraphPad Prism Software Inc., La Jolla, CA) was used to perform these analyses. Confidence p-levels above columns indicated by asterisks are given with annotation as ns - non significant, i.e. *p < 0.05, **p < 0.01, ***p < 0.001.

## Additional Information

**How to cite this article**: Blazevits, O. *et al.* Galectin-1 dimers can scaffold Raf-effectors to increase H-ras nanoclustering. *Sci. Rep.* **6**, 24165; doi: 10.1038/srep24165 (2016).

## Supplementary Material

Supplementary Information
