# Supplementary material for: Galectin-1 dimers can scaffold Raf-effectors to increase H-ras nanoclustering
Source: Sci Rep. 2016 Apr 18;6:24165. doi: 10.1038/srep24165 (PMC4834570; doi:10.1038/srep24165)
Supplement: Supplementary Information [file srep24165-s1.pdf]

# Supplementary information

## Galectin-1 dimers can scaffold Raf-effectors to increase H-ras nanoclustering

**Authors**

Olga Blazevits*[1], Yonatan G Mideksa*[1], Maja Solman[1], Alessio Ligabue[1], Nicholas Ariotti[2], Hosein Nakhaeizadeh[3], Eyad K Fansa[4], Anastassios C Papageorgiou[1], Alfred Wittinghofer[4], Mohammed R Ahmadian[3], Daniel Abankwa[1]

*equal contributions

**Author affiliations**

[1] Turku Centre for Biotechnology, Åbo Akademi University, Tykistökatu 6B, 20520 Turku, Finland; [2] Institute for Molecular Bioscience, The University of Queensland, St. Lucia, QLD 4072, Australia; [3] Institute of Biochemistry and Molecular Biology II, Medical Faculty, Heinrich-Heine-University, Düsseldorf, Germany; [4] Max Planck Institute for Molecular Physiology, 44227 Dortmund, Germany

**Contact for correspondence:**

Daniel Abankwa, daniel.abankwa@btk.fi, +358-333 6969

**Supplementary Table 1. Gal-1 and C-Raf-RBD mutants in this study**

First column specifies the mutated protein or protein domain, the second column gives the name used for the mutant in the manuscript and the last column the actual amino acid residues mutated in the sequence of the full length protein.

| Protein | Mutant name | Mutated amino acid residues |
|---|---|---|
| **C-Raf-RBD** | RBD-N3A | K109A,W114A,T116A |
| | RBD-C3A | W114A,T116A,L121A |
| | RBD-6A | K109A,W114A,T116A,L121A,E124A,L126A |
| | RBD-D117A | D117A |
| | RBD-D113A,D117A | D113A,D117A |
| | RBD-D117R | D117R |
| | RBD-R100D | R100D |
| **Galectin-1** | Gal-1-S63A | S63A |
| | Gal-1-S63A,D65A | S63A,D65A |
| | Gal-1-Q73A | Q73A |
| | Gal-1-K29T | K29T |

## Supplementary Figure legends

### Supplementary Figure 1.

(**A**) FLIM-FRET measurements of mGFP-H-rasG12V and mRFP-Gal-1 complexation in BHK21 cells with or without 5 μM compactin treatment. While compactin treatment reduced the FRET significantly, FRET was still in agreement with a complexation between H-rasG12V and Gal-1 in the cellular cytoplasm. Plotted values represent the mean ± SEM and numbers inside the bars correspond to the total number of cells imaged in each case. Statistical differences between compactin treated and untreated cells were calculated as described in **Methods** from three independent biological repeats (***, $p < 0.001$).

(**B**) Interaction between non-farnesylatable mGFP-H-rasG12V-C186S and mRFP-tagged Gal-1, as well as mGFP-H-rasG12V and putative farnesyl-pocket mutant Gal-1-K29T studied using FLIM-FRET in HEK293-EBNA cells transiently expressing indicated constructs. As compared to the positive control H-rasG12V and C-Raf-RBD, the binding between H-rasG12V-C186S and C-Raf-RBD was decreased due to the loss of H-ras plasma membrane anchorage, where H-rasG12V exhibits a higher recruitment efficiency [1]. However, significant FRET between H-rasG12V-C186S and Gal-1 was still retained. In addition, the mutation K29T in Gal-1 could not disrupt the complexation between H-rasG12V and Gal-1, as the FRET compared to the (FRET-) control sample was statistically significant (not indicated). Samples coexpressing mGFP and mRFP served as a FRET control.

(**C**) Schematic representation of ACP-tag protein expression and fluorescence labelling for *in vitro* experiments. Recombinantly expressed and purified proteins were used for studies in solution. The protein expression plasmid pQE-A1 was constructed on the basis of commercial pQE-30 Xa vector (Qiagen). The pQE-A1-

plasmid has BglII and KpnI restriction sites for sub-cloning of different genes of interest. The final fusion protein consists of four functional elements: 1) the N-terminus contains 6-His affinity tag for purification on a nickel chelate column; 2) the PreScission protease recognition site for a site-specific protease cleavage (indicated by the scissors); 3) the A1-tag is an alternative acyl carrier protein (ACP)-tag (NEB) with a short amino acid sequence (12 aa) used for selective fluorescent labelling; 4) the final element is the sequence of the protein of interest. ACP labelling reaction: ACP synthase (4′-phosphopantetheinyl transferase) catalyzes the covalent transfer of substituents from derivatized coenzyme A (CoA) to A1-tagged fusion proteins in solution. The A1-tag is a small tag (8 kDa) based on the acyl carrier protein (ACP) that allows the specific, covalent attachment of virtually any molecule to a protein of interest. Substrates for labelling are derivatives of coenzyme A (CoA). In the labelling reaction, ACP Synthase covalently attaches the substituted phosphopantetheine group of CoA to a serine residue on the A1-tag.

(**D**) Hemagglutination assay with mouse red blood cells to test and compare purified A1-Gal-1 and His-A1-Gal-1 carbohydrate-binding activity. The graph depicts mean maximum OD values versus 1:2 serial dilutions of proteins combined with 30 μl red blood cell suspension. The transmission characteristics of a suspension of red blood cells are determined by the combined effect of the absorption by intracellular hemoglobin and the scattering from the cells. Both these parameters are affected by the shape and volume of the cells. A decrease in OD from left to right correlates with spread out red blood cells (positive outcome). Concanavalin A is a positive control. The high concentration ranges where the two curves meet indicates overlapping hemagglutination properties of Gal-1 variants with those of concanavalin A samples[2]. On the other hand, at low concentration ranges where there is no galectin-induced

hemagglutination, the two curves stay separated unlike the positive outcome from concanavalin A samples. Data points are means ± SEM from two individual biological repeats.

(**E**) Representative western blots from HEK293-EBNA cellular lysates expressing indicated constructs immobilized on lactose agarose beads. Cells transfected with the carbohydrate-binding deficient mutant (mRFP-Gal-1-N47D,W69L) and untransfected cells serve as controls. The load, flow-through (unbound fraction), and elution (retained fraction) were analysed using a Gal-1 antibody. Right panel shows repeated experiment with higher amount of load. Arrow points to the position of bands corresponding to mRFP-labelled Gal-1 proteins (between 37 kDa - 50 kDa).

(**F**) In vitro experiment FRET-controls. Samples of fluorescent labels CoA-488 or ATTO-488 (actual dye in CoA-488) as FRET donors and CoA-547 or DY-547 (actual dye in CoA-547), as well as mRFP-tagged C-Raf-RBD or Gal-1 as FRET acceptors were analysed by FLIM-FRET microscopy. Each control sample was a 1:1 mixture of fluorescent labels with proteins each at 1 μM. The sample set shows that the CoA-547 dye exhibits significant background FRET, due to the CoA-moiety, as the free dyes do not show this FRET. On the other hand, CoA-488 and ATTO-488 FRET samples show comparable FRET.

(**A, B, F**) Plotted values correspond to the mean ± SEM of at least three independent biological experiments. Numbers inside the bars indicate total number of cells or fields of view imaged. The **Methods** section describes the statistical comparisons as indicated or against the parent/ non-mutant or untreated conditions (**, $p < 0.01$; ***, $p < 0.001$).

**Supplementary Figure 2.**

(**A**) Schematic representation of Raf-paralogs with conserved regions CR1-3 indicated. CR1 contains the Ras binding domain (RBD) and the cysteine-rich domain (CRD), which are both required for membrane recruitment. CR2 contains regulatory binding sites. CR3 is the catalytic domain.

(**B**) The nanoclustering-FRET response of H-rasG12V with or without silencing of A-, B- or C-Raf paralogs combined with Gal-1 overexpression. Raf paralogs in HEK293-EBNA cells were silenced for 48 h. Then 24 h after the transfection with Raf siRNA cells were transiently co-transfected with pmGFP-H-rasG12V, pmCherry-H-rasG12V and pcDNA3-Gal-1, as indicated. H-rasG12V nanoclustering without silencing and Gal-1 overexpression was used as a control (highlighted graph). FRET between fluorescent proteins mGFP and mCherry is a negative control. Plotted values represent the mean ± SEM and numbers inside the bars correspond to the total number of cells imaged in each case measured in three independent biological repeats. Statistical significance between treated samples and marked control (dark grey) was determined as described in **Methods** (ns, nonsignificant; ***, $p < 0.001$).

(**C**) Multiple sequence alignment of human A-, B- and C-Raf- and PI3Kα- RBDs. **Top panel**: Shown here are the Ras-binding region, which harbour major binding residues with Ras, and candidate C-Raf-RBD/ Gal-1 interface residues at distance less than 5Å (red arrowheads) from Gal-1. Key residues that abolished RBD/ Gal-1 complex formation in experiments after they were mutated to alanine are marked with asterisks. Nuclear import (NLS) and export (NES) sequence stretches with high activity score (cNLS mapper) are marked in light yellow and blue shaded boxes, respectively. **Bottom panel**: Optimal pairwise alignment of C-Raf- and PI3Kα-RBDs

identifies a conserved residue, which computational modelling and experimental data suggest as being central to complex formation between Gal-1 and the C-Raf-RBD.

**(D)** C-Raf-RBD directly binds GST-Gal-1. Pull-down experiments were performed by mixing bacterially purified C-Raf-RBD and GST-Gal-1 immobilized on glutathione sepharose beads. GST was used as a control. Proteins retained on the beads (output) were resolved by SDS-PAGE Laemmli buffer and processed for SDS-PAGE gel, which was stained using coomassie brilliant blue. Standard proteins were used as molecular weight markers.

**Supplementary Figure 3.**

**(A)** Complexation between mGFP-tagged Gal-1 and mRFP-tagged C-Raf-RBD measured by FLIM-FRET in HEK293-EBNA cells (**left**). Nanoclustering-FRET studied in HEK293-EBNA co-expressing mGFP- and mCherry-tagged H-rasG12V (**right**). Cells were treated for two hours with DMSO control or 20 μM of the thiodigalactoside Gal-1/-3 inhibitor Di-(3-deoxy-3-(4-((butylamino)carbonyl)-1H-1,2,3- triazol-1-yl)-β-D-galactopyranosyl)sulfane, IC-26-177-1[3], or were left untreated. This compound has a 10x higher selectivity towards Gal-3 than Gal-1 (Dr. Ulf Nilsson, Galecto Biotech, personnel communication). Interaction between mGFP and mRFP only served as a FRET control.

(**B**) FRET binding data between C-Raf-RBD or PI3Kα-RBD and Gal-3. Fluorescence lifetime of HEK293-EBNA cells transiently expressing mCit-tagged Gal-3 and mRFP-tagged RBDs was measured using FLIM-FRET.

**(C)** Sequence alignment of *Rattus norvegicus* Gal-1 and human Gal-3. Critical residues recognizing the carbohydrate ligand are highly conserved among mammalian

galectins (green shaded boxes). Displayed above the sequence by red arrowheads are 'hot spot' interfacial residues identified in this study. Corresponding predicted key residues that facilitate Gal-1/ RBD complex formation were mutated to alanine (asterisks). A bipartite NLS stretch is centred at the C-terminal end of Gal-1 (light yellow shaded box).

(**D**) Computational representation of carbohydrate ligand-bound, monomeric Gal-1 (*1GZW*)[4] and C-Raf-RBD (*1RFA*)[5] complex from preliminary low energy molecular docking pose. Shown here are interfacial predicted candidate residues with Gal-1's S63 and D65 side-chains forming hydrogen bonds with RBD's D113 and W114, respectively. RBD's D117 residue makes hydrogen bonds with T71 from Gal-1. Mutant residues used in this study for both proteins in the complex are marked here by asterisks. Numbering of residues is according to sequences deposited in UniProt (P09382 – Gal-1_*Homo sapiens,* P04049 – C-Raf_*Homo sapiens).* The loop that undergoes major conformational and stereochemical changes (loop4) between apo- and liganded Gal-1 is coloured orange.

(**E, F**) Representative confocal images of HEK293-EBNA cells co-expressing indicated pmGFP-Gal-1- and pmRFP-C-Raf-RBD-constructs to verify localization of Gal-1/ RBD-interfacial mutants (**see Supplementary Table 1**). Columns represent imaged fluorescent channels, appropriate for the indicated construct. Nucleus was DAPI stained. Scale bar is 5 μm.

(**G**) FRET binding data between Gal-1 interfacial mutants derived from the Gal-1/ C-Raf-RBD pose shown in (**D**). Fluorescence lifetimes of HEK293-EBNA cells transiently expressing GFP-tagged C-Raf-RBD and RFP-tagged Gal-1 mutants were measured using FLIM-FRET. Samples coexpressing mGFP and mRFP only served as a negative control.

(**H**) FLIM-FRET binding data of mGFP-tagged H-rasG12V with mRFP-tagged C-Raf-RBD or the candidate interfacial mutant C-Raf-RBD-R100D in HEK293-EBNA cells. Significant loss of FRET indicates undesired changes in RBD affinity/ folding.

(**A, B, G, H**) Cells from three independent biological experiments were analysed and numbers inside the bars correspond to the total number of imaged cells. Plotted values correspond to the mean ± SEM. Statistical analysis of the samples against appropriate controls was performed as described in **Methods** (ns, nonsignificant; ***, $p < 0.001$). Note that non-control sample FRET-values were all significantly different from the (FRET-) control sample.

**Supplementary Figure 4.**

(**A**) Dimerization of wt Gal-1 with its dimer interface mutants measured by FLIM-FRET in HEK293-EBNA cells transiently co-expressing mRFP-tagged wt Gal-1 and mGFP-tagged wt Gal-1, Gal-1-V6D or N-Gal-1 as indicated. The schematic representation on the left illustrates the FRET-pair. FRET between wt Gal-1 and N-Gal-1 showed a significant decrease as compared to wt Gal-1/ wt Gal-1 consistent with a reduced dimerization potential of the mutant, while this was not the case for the Gal-1-V6D mutant.

(**B**) Western blot showing dimer/monomer equilibrium of Gal-1 in HEK293-EBNA cell lysates. Cells were transfected with mRFP-Gal-1 or mRFP-N-Gal-1 and lysates were run on the native tricine PAGE. The different forms of Gal-1 were analysed using human Gal-1 antibody. Arrows point to the position of bands corresponding to monomeric and dimeric Gal-1 and dimerization- deficient Gal-1 mutant, N-Gal-1 (37-40kDa).

(**C**) FRET binding data on the C-Raf-RBD with dimer interface mutants of Gal-1 in HEK293-EBNA cells co-expressing mGFP-tagged C-Raf-RBD and mRFP-tagged wt Gal-1, Gal-1-V6D or N-Gal-1 as indicated.

(**D**) Complexation of mGFP-K-rasG12V or mGFP-N-rasG12V with mRFP-Gal-1 in BHK21 cells, with or without 5 μM compactin treatment, measured by FLIM-FRET.

(**A, C, D**) The fluorescence lifetime of the donor was measured using FLIM-FRET. Numbers inside the bars indicate the total number of the imaged cells. Statistical analysis was performed as described in **Methods** against parent or untreated control samples (ns, nonsignificant; *, $p < 0.05$; ***, $p < 0.001$). Samples coexpressing mGFP and mRFP represent the FRET control. Note that non-control sample FRET-values were all significantly different from the (FRET-) control sample.

(**E**) Western blot showing endogenous levels of Gal-1 in HEK293-EBNA and BHK21 cell lysates. β-actin was used as a loading control. The right panel shows overexposed bands from the same blotting membrane to better visualize the low intensity band in the HEK293-EBNA sample.

**Supplementary references**

1. Guzmán, C. *et al.* The efficacy of Raf kinase recruitment to the GTPase H-ras depends on H-ras membrane conformer-specific nanoclustering. *Journal of Biological Chemistry* **289,** 9519–9533 (2014).

2. Shu, Z. *et al.* Expression, purification and characterization of galectin-1 in Escherichia coli. *Protein Expr. Purif.* **99,** 58–63 (2014).

3. Salameh, B. A., Cumpstey, I., Sundin, A., Leffler, H. & Nilsson, U. J. 1H-1,2,3-

triazol-1-yl thiodigalactoside derivatives as high affinity galectin-3 inhibitors. *Bioorg. Med. Chem.* **18,** 5367–5378 (2010).

4. López-Lucendo, M. F. *et al.* Gene design, expression, crystallization and preliminary diffraction analysis of two isolectins from the fungus Coprinus cinereus: a model for studying functional diversification of galectins in the same organism and their evolutionary pathways. *Acta Crystallogr. D Biol. Crystallogr.* **60,** 721–724 (2004).
5. Emerson, S. D. *et al.* Solution structure of the Ras-binding domain of c-Raf-1 and identification of its Ras interaction surface. *Biochemistry* **34,** 6911–6918 (1995).

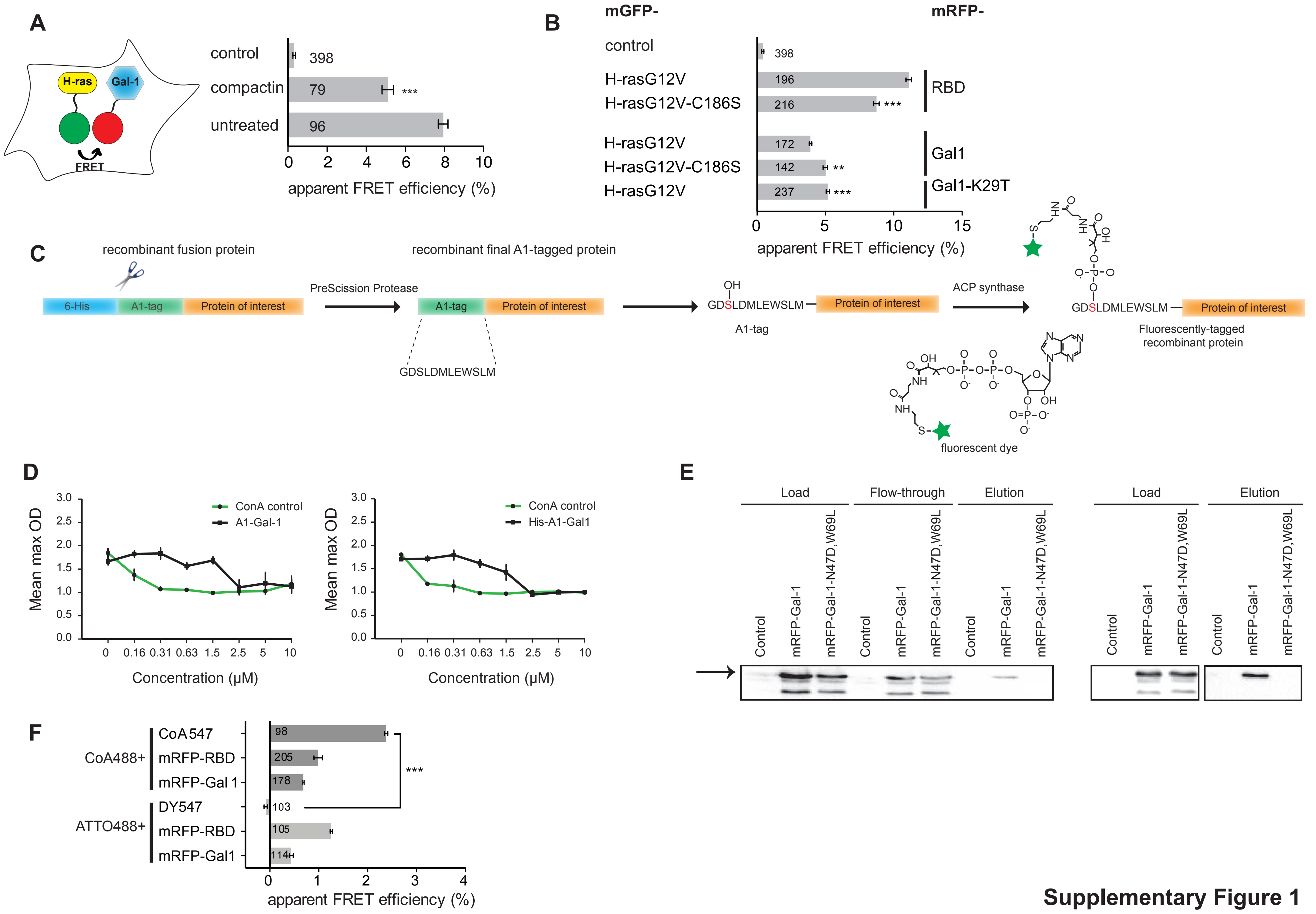


Supplementary Figure 1

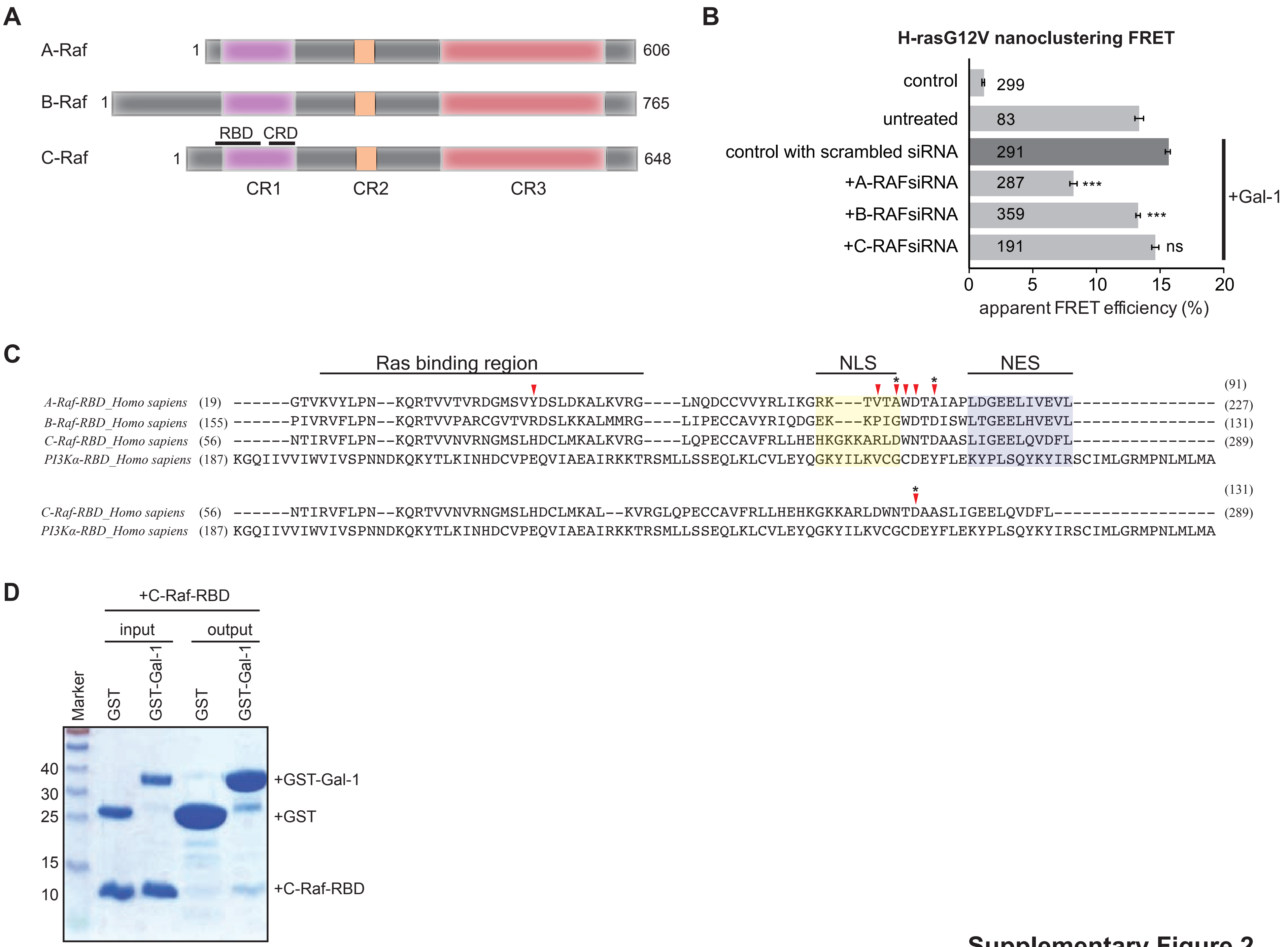


**Supplementary Figure 2**

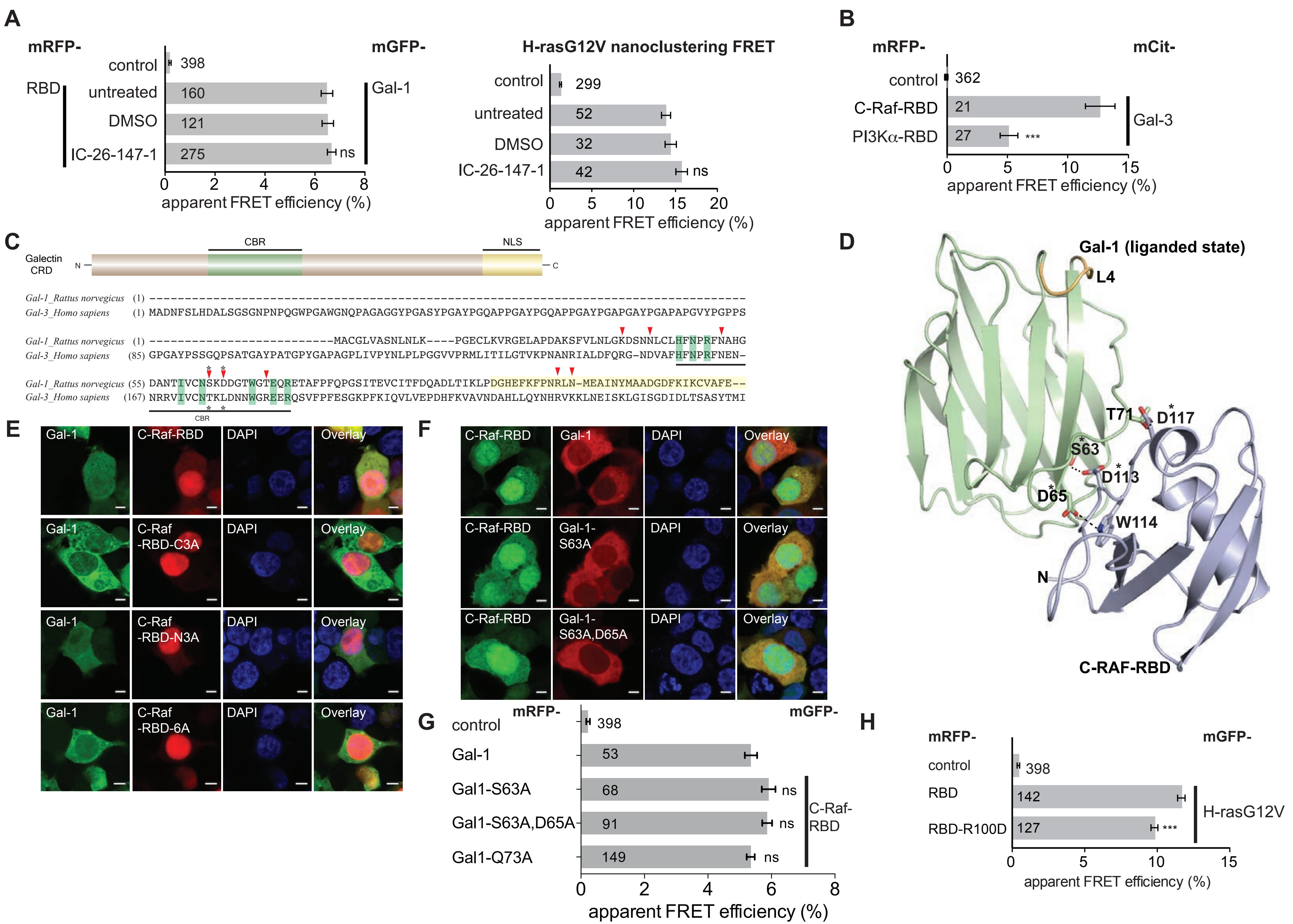


**Supplementary Figure 3**

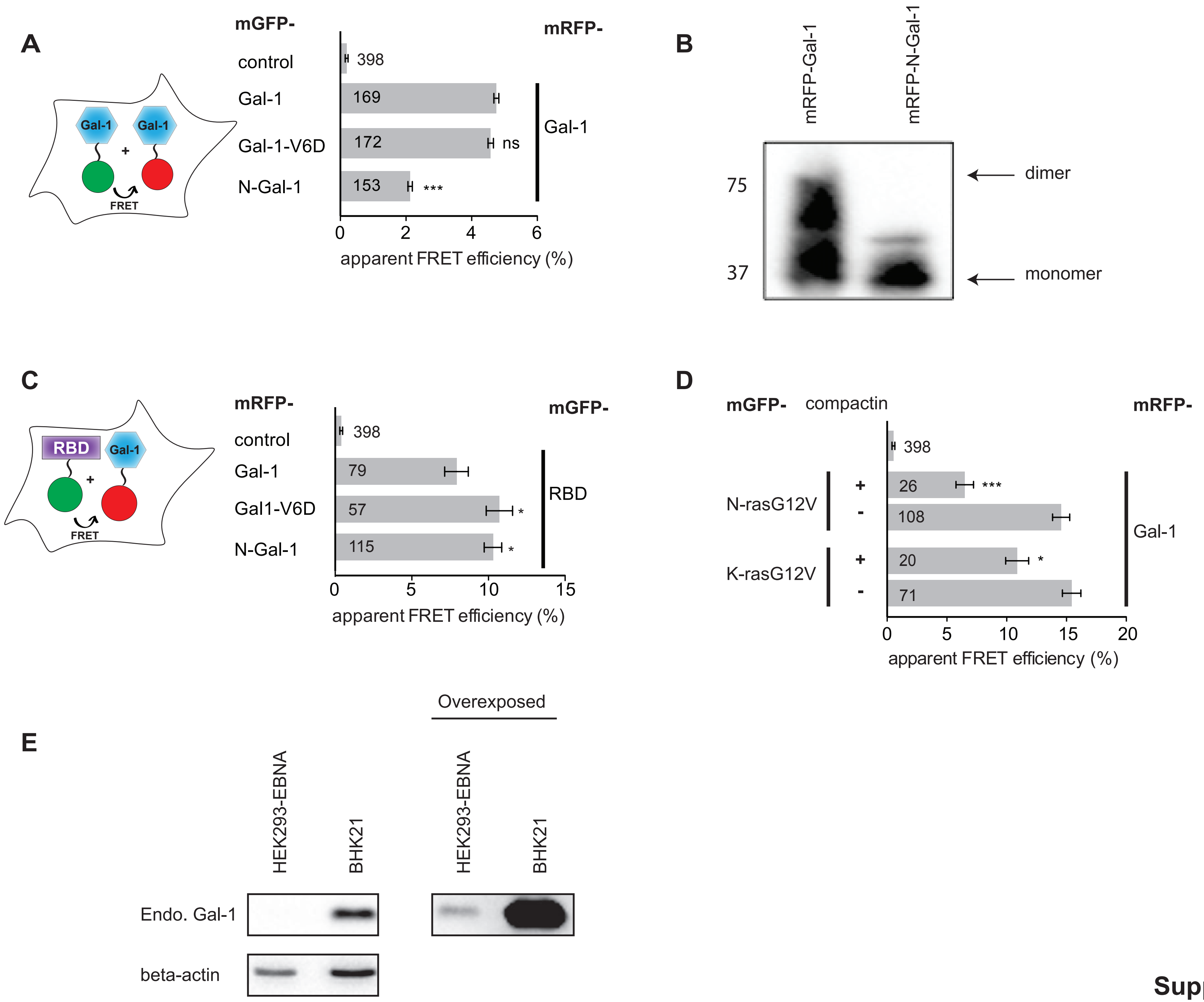


**Supplementary Figure 4**
